# Structures and Anti-Inflammatory Evaluation of Phenylpropanoid Derivatives from the Aerial Parts of *Dioscorea polystachya*

**DOI:** 10.3390/ijms231810954

**Published:** 2022-09-19

**Authors:** Baixiang Cai, Xinyin Cai, Tao Xu, Jutao Wang, Yang Yu

**Affiliations:** 1 School of Pharmacy, Anhui University of Chinese Medicine, Hefei 230012, China; 2 Institute of Medicinal Chemistry, Anhui Academy of Chinese Medicine, Hefei 230012, China; 3 The MOE Key Laboratory of Standardization of Chinese Medicines and the SATCM Key Laboratory of New Resources and Quality Evaluation of Chinese Medicines, Shanghai University of Traditional Chinese Medicine, Shanghai 201203, China; 4 Institute of Chinese Materia Medica, Shanghai University of Traditional Chinese Medicine, Shanghai 201203, China; 5 Department of Biological and Pharmaceutical Engineering, West Anhui University, Luan 237012, China; 6 Anhui Province Key Laboratory of Research & Development of Chinese Medicine, Hefei 230012, China; 7 Institute for Pharmacodynamics and Safety Evaluation of Chinese Medicine, Anhui Academy of Chinese Medicine, Hefei 230012, China

**Keywords:** phenylpropanoid, *Dioscorea polystachya*, anti-inflammatory activity

## Abstract

Seven undescribed phenylpropanoid constituents, including three new bibenzyl derivatives (**1**–**3**) along with four new benzofuran stilbene derivatives (**4**–**7**), were isolated from the aerial parts of *Dioscorea polystachya*. The structures of these compounds were elucidated using a combination of spectroscopic analyses, including UV, IR, HRESIMS, 1D, and 2D NMR. Further, all the compounds were evaluated on the anti-inflammatory activity for their inhibition of nitric oxide (NO) production by RAW 264.7 macrophages cells, and some of them (**1**–**3** and **6**) displayed inhibitory activity with IC_50_ values in the range of 9.3–32.3 μM. Moreover, compound **3** decreased the expression of iNOS in Western blot analysis, suggesting compound **3** is mediated via the suppression of an LPS-induced NF-κB inflammasome pathway.

## 1. Introduction

The genus of *Dioscorea* is a perennial herbaceous and monocotyledon plant, and has large roots and/or rhizomes [1,2,3,4]. *Dioscorea* plants are widely distributed in China. The *D. polystachya* tuber has been edible with a history of more than 3000 years in China, and it is also an important traditional Chinese herbal plant. It is used for the treatment of atherosclerosis, coughing with lung heat, hypertension, pyretic stranguria, anthracia, hyperglycemia, coronary heart disease, swelling, gastric ulcers, and sprains [5,6,7,8,9]. Previous phytochemistry investigations focused on the genus of *Dioscorea* and resulted in the isolation of bibenzyls, phenanthrenes, biphenanthrenes, lignans, steroidal saponins, flavonoids, and polysaccharides [10,11,12].

**Figure 1:**
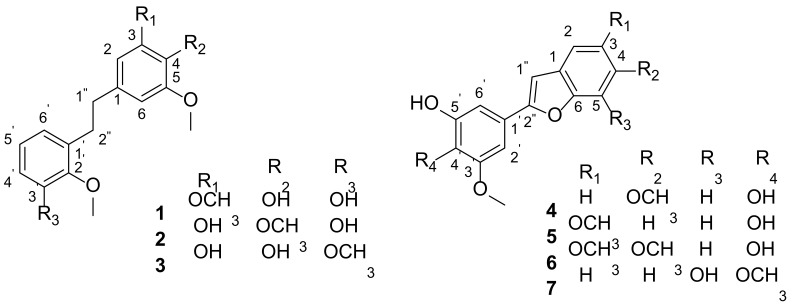
The chemical structures of compounds **1**–**7** obtained from the aerial parts of *D. polystachya*.

However, there is still a deficiency of bioactive compounds from the aerial parts *D. polystachya*. With our ongoing research of chemical constituents and biological activity with herbal medicine [13,14,15], phytochemical studies of the aerial parts of *D. polystachya* were carried out in this research in consideration of explore anti-inflammatory ingredients. Here, seven undescribed phenylpropanoid constituents, including three new bibenzyl compounds (**1**–**3**) along with four new benzofuran stilbene compounds (**4**–**7**), were isolated from the aerial parts of *D. polystachya* (Figure 1) and their chemical structures were elucidated based on UV, IR, NMR, and HRESIMS spectroscopic data. Compound **3**, a bibenzyl compound, exhibits strong anti-inflammatory effect, and it should inhibit the inflammatory signaling mechanism of NF-κB pathways. In this report, their isolation, structural characterization, and potential anti-inflammatory activities are described.

## 2. Results

### 2.1. Isolation of New Compounds **1**–**7** from the Aerial Parts of D. polystachya

The fresh aerial parts of *D. polystachya* (5 kg) were extracted three times with methanol to give a crude extract, then the extract was suspended in water and extracted with EtOAc, which were subjected to MCI, C_18_ column chromatography, MPLC, preparative HPLC, and preparative TLC to obtain seven new phenylpropanoid compounds **1**–**7** (Figure 1).

### 2.2. Structure Identification of New Compounds **1**–**7**

**Figure 2:**
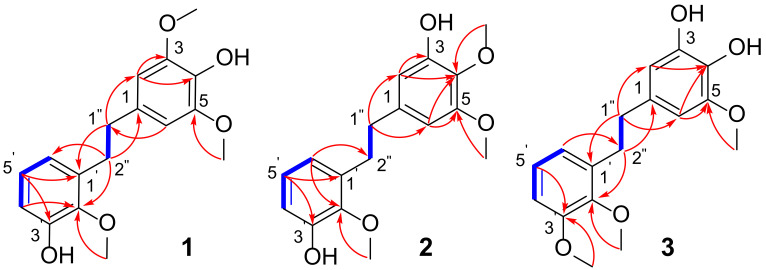
^1^H-^1^H COSY (-) and key HMBC (→) correlations of compound **1**–**3**.

Compound **1**, a faint yellow solid powder, afforded a molecular formula of C_17_H_20_O_5_ based on the (-)-HRESIMS ion peak at *m/z* 303.1238 [M-H]^−^ (calcd. for 303.1238) and ^13^C NMR data (Figure S1), corresponding to eight degrees of hydrogen deficiency. The UV spectrum indicated absorption peaks at 272 nm for benzanyl. The IR spectrum displayed typical absorption peaks attributed to broad hydroxyl (3425 cm^−1^), aromatic ring (1613, 1517 cm^−1^), and methine (1469 cm^−1^) functionalities. Its ^1^H NMR spectrum exhibited a typical signal for a symmetrical structure [*δ*_H_ 6.37 (2H, s, H-2/6), 3.75 (6H, s, 3/5-OCH_3_)], one set of ABC aromatic protons at *δ*_H_ 6.59 (1H, dd, *J* = 7.8, 1.4 Hz), 6.67 (1H, dd, *J* = 7.8, 1.4 Hz), and 6.80 (1H, t, *J* = 7.8 Hz), one additional methoxy group (*δ*_H_ 3.70, 3H, s), and two methine protons (*δ*_H_ 2.78, 2H, m; *δ*_H_ 2.84, 2H, m). The ^13^C NMR data (Table 1) and HSQC spectrum showed 17 carbon resonances, comprising seven nonprotonated carbons (*δ*_C_ 134.1, 149.0 × 2, 134.5, 136.6, 147.2, 151.0), five methines (*δ*_C_ 106.7 × 2, 115.4, 125.0, 122.1), two methylenes (*δ*_C_ 38.1, 33.2), and three methoxy groups (*δ*_C_ 56.6 × 2, 60.8). The NMR data of **1** (Table 1) were suggestive of those of a bibenzyl compound [16]. The COSY correlations of H-4 to H-5, and H-5 to H-6, combined with the ABC aromatic proton signals, indicated the presence of a 1,2,3-trisubstituted benzene in **1** (Figure 2). The HMBC correlations from H-2/6 to C-3/5 (*δ*_C_ 149.0) and 3/5-OCH_3_ to C-3/5 indicated that the two symmetrical methoxy groups were linked at C-3/5. The location of the additional methoxy group (*δ*_H_ 3.70, *δ*_C_ 60.8) at C-2′ was confirmed by the HMBC cross-peak of H-2″ with C-2′ (*δ*_C_ 147.2). The ROESY correlations were consistent with the above speculation (Figure S8). Thus, the structure of compound **1** was established as shown (Figure 1) and named diosbiben A.

Compound **2** was obtained as a yellowish solid powder. Its molecular formula had the same HRESIMS data as compound **1**, indicating they were isomers. The NMR data (Figure S2) showed that compounds **1** and **2** had similar structures. Nevertheless, they have different polars in HPLC analysis. The 1D NMR spectra (Table 1) exhibited the following: one set of aromatic protons at *δ*_H_ 6.27 (1H, d, *J* = 1.6 Hz), 6.33 (1H, d, *J* = 1.6 Hz), with corresponding carbon signals at *δ*_C_ 139.5 (C-1), 110.1 (C-2), 151.2 (C-3), 135.8 (C-4), 154.2 (C-5), and 105.2 (C-6), indicating an unsymmetrical tetrasubstituted benzene moiety. In the HMBC spectrum (Figure 2), the correlations from H-2/6 to C-4 revealed that the methoxy group was linked to C-4. The HMBC correlations from H-2 to C-3 (*δ*_C_ 151.2) indicated that a hydroxy group was linked to C-3. Therefore, compound **2** was determined as 4,5,2′-trimethoxy-3,3′-dihydroxy-bibenzyl and named diosbiben B.

Compound **3**, a faint yellow solid, had the molecular formula of C_17_H_20_O_5_ from HRESIMS data and an unsaturation of 8. The UV spectrum of **3** was similar to **1** and **2**. Typical absorption peaks attributed to broad hydroxyl (3290 cm^−1^), aromatic ring (1612, 1518 cm^−1^), and methine (1474 cm^−1^) functionalities were observed in the IR spectrum. Its molecular formula and NMR data (Figure S3) were similar to that of compounds **1** and **2**, indicating that they were possible isomers and also were bibenzyl compounds. The HMBC correlations from H-2/6 to C-4 (*δ*_C_ 133.1) indicated a hydroxy group was linked to C-4. One methoxy group was linked to C-3′, which was also demonstrated by the following correlations in the HMBC spectrum: H-5′ to C-3′ (*δ*_C_ 154.0); 3′-OCH_3_ to C-3′. Finally, the structure of **3** was identified as 5,2′,3′-trimethoxy-3,4-dihydroxy-bibenzyl and named diosbiben C.

**Figure 3:**
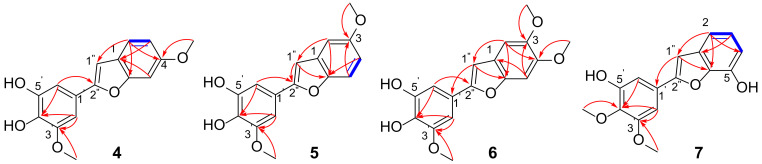
^1^H-^1^H COSY (-) and key HMBC (→) correlations of compound **4**–**7**.

Compound **4** possesses a molecular formula of C_16_H_14_O_5_ (ten indices of hydrogen deficiency), as deduced from HRESIMS (*m/z* 285.0768 [M-H]^-^; calcd 285.0768) and ^13^C NMR data (Figure S4). Typical long conjugate absorption peaks were observed at 285 nm in the UV spectrum. The IR spectrum revealed typical absorption peaks for hydroxyl group (3409 cm^−1^), and aromatic ring (1622, 1515 cm^−1^) functionalities. An analysis of the ^1^H and ^13^C NMR spectra (Table 2 and Table 3) showed characteristic signals for a set of ABX aromatic signals (*δ*_H_ 7.38, d, *J* = 8.5 Hz, *δ*_C_ 121.6; *δ*_H_ 6.82, dd, *J* = 8.5, 2.0 Hz, *δ*_C_ 112.6; *δ*_H_ 7.07, d, *J* = 2.0 Hz, *δ*_C_ 96.6). The ^13^C NMR and HSQC data revealed the presence of eight quaternary carbons (including six oxygenated), six sp2 methines, and two methoxy carbons. The above data indicated that **4** was a benzofuran stilbene derivative [17], which also confirmed by the HMBC correlations (Figure 3) of H-2′/6′ to C-2″, H-2 to C-1″/6, and H-3/5 to C-1. The HMBC correlations of H-2/4-OCH_3_ to C-4 (*δ*_C_ 159.2) suggested a methoxy group connect to C-4 in benzofuran. Another methoxy group was found to be linked to the aromatic ring (2″-phenyl) at C-3′ from the HMBC correlations of H-2′/3′-OCH_3_ and C-3′ (*δ*_C_ 149.9). Consequently, **4** was concluded to be 4,3′-dimethoxy-4′,5′-dihydroxy-2″-phenylbenzofuran and named diosbenfura A.

Compound **5** was assigned the molecular formula C_16_H_14_O_5_ as determined by its HRESIMS (*m/z* 285.0767 [M-H]^−^; calcd 285.0768) and ^13^C NMR data (Figure S5). The molecular formula and NMR spectra of compound **5** were similar to those of compound **4**, indicating that they were possible isomers. NMR data indicated a difference position of the 3-OCH_3_ group. The HMBC correlations from H-5/3-OCH_3_ to C-3 indicated that methoxy group was linked to C-3 not C-4 in **5**. Similarly, the structure of **5** was established as 3,3′-dimethoxy-4′,5′-dihydroxy-2″-phenylbenzofuran and named diosbenfura B.

Compound **6** was isolated as a yellow solid powder with a molecular formula of C_17_H_17_O_6_ (index of hydrogen deficiency of 10) as determined by HRESIMS (*m/z* 315.0875 [M-H]^−^, calculated 315.0874) and further supported by ^1^H, ^13^C, and edited-HSQC NMR data (Figure S6). The IR spectrum of **6** displayed typical absorption peaks attributed to hydroxyl group (3428 cm^−1^), and aromatic ring (1618 cm^−1^, 1486 cm^−1^) functionalities. Compound **6** showed NMR signals indicative sharing structural similarities with **4** and **5** (Table 2 and Table 3). The NMR and HRMS data for **6** suggested that the presence of one additional methoxy group relative to **4** and **5**. HMBC correlations from H-5/3-OCH_3_ to C-3 (*δ*_C_ 147.9), H-1/4-OCH_3_ to C-4 (*δ*_C_ 149.1) confirmed the connectivity of the two methoxy groups in benzofuran. Accordingly, compound **6** was determined to be 3,4,3′-trimethoxy-4′,5′-dihydroxy-2″-phenylbenzofuran and named diosbenfura C.

Compound **7** was obtained as a yellow solid powder. It showed high similarity to compounds **4** and **5**, including a molecular formula of C_16_H_14_O_5_ (HRESIMS *m/z* 285.0767 [M-H]^−^, calculated 285.0768) and NMR data (Figure S7), which were indicative of a benzofuran stilbene analogue. The IR spectrum showed absorption bands for hydroxyl group at 3414 cm^−1^ and aromatic ring at 1605 and 1494 cm^−1^. ^1^H NMR data of **7** clearly displayed a set of ABC aromatic proton signals at *δ*_H_ 6.58 (1H, d, 7.8), 7.06 (1H, t, 7.8), 6.98 (1H, d, 7.8). Two methoxy group positions were assigned further by interpretation of the HMBC correlations from H-2′/6′/4′-OCH_3_ to C-4′ (*δ*_C_ 138.1), H-2′/3′-OCH_3_ to C-3′ (*δ*_C_ 154.9). Moreover, compound **7** was designated as 3′,4′-dimethoxy-5,5′-dihydroxy-2″-phenylbenzofuran and named diosbenfura D.

### 2.3. Inhibitory Effects of New Compounds **1**–**7** on NO Production of LPS-Activated RAW 264.7 Cells

All the isolates were tested for their effects on nitric oxide (NO) production inhibition in lipopolysaccharide (LPS)-activated RAW 264.7 cells. In order to exclude the inhibition of NO production caused by cytotoxicity, cell viability was evaluated by the MTT method. Results revealed that no obvious cytotoxicity (over 75% cell survival) for most of compounds at concentrations up to 50 μM was observed. The results shown in Table 4, most of the isolated compounds (**1**–**3**, **6**) that displayed NO inhibitory activity (positive control: Aminoguanidine hydrochloride, IC_50_ 19.2 ± 0.78 μM). The NO inhibitory activity of compounds **1**–**3** (IC_50_ 9.3–32.3 μM) versus **6** (IC_50_ 24.1 μM) suggested the bibenzyl compounds were more active than benzofuran stilbene compounds. The position of substituent may play a significant role in mediating the activities.

### 2.4. Inhibitory Effects of New Compound **3** on LPS-Enhanced Inflammatory Mediators

**Figure 4:**
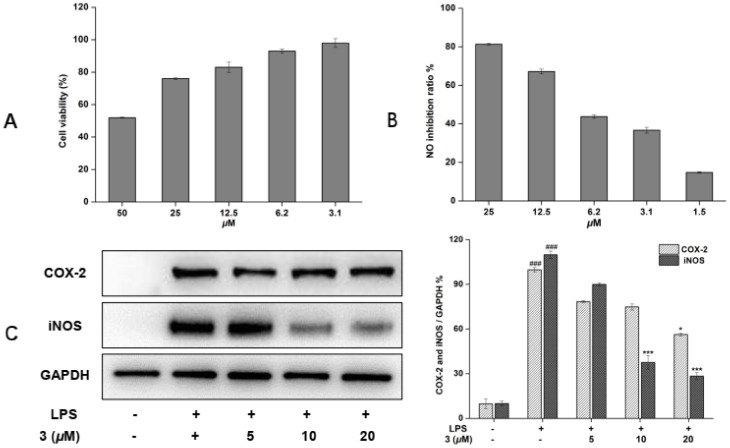
Effects of compound **3** on cell viability (50–3.1 μM) (**A**). NO inhibitory activity (25–1.5 μM) in RAW 264.7 cells (**B**). iNOS and COX-2 protein levels were detected by a Western blot assay (**C**). *** *p* < 0.001, ^###^ *p* < 0.001, * *p* < 0.01 compared to the LPS-treated group.

Since compound **3** displayed the strongest inhibition on NO produce of all isolated compounds, so it was chosen for mechanistic research. Compound **3** displayed no cytotoxicity at concentrations up to 25 μM (Figure 4A); thus, this concentration was used in subsequent experiments. As shown in Figure 4B, the significant NO production inhibitory effect of **3** with an IC_50_ of 9.3 ± 1.03 μM was observed. As is well known, the production of NO is closely related to the key proteins iNOS and COX-2 [18]. Therefore, the expression levels on these proteins were detected by Western blotting. As shown in Figure 4C, a mild down-regulation of COX-2 expression was observed, but the levels of iNOS were inhibited in the presence of **3** at 10 μM. Based on these observations, it was inferred that **3** exhibited inhibitory effects on NO production may by means of suppression of iNOS in LPS-induced RAW 264.7 macrophages.

## 3. Discussion

The biological activities of *D. polystachya*, which have not been fully demonstrated. Moreover, few studies have been conducted with the metabolites of the aerial parts of *D. polystachya*. In the current study, the secondary metabolites of the aerial parts of *D. polystachya* were investigated by chromatographic purification and interpretation of spectroscopic data to generate seven new phenylpropanoid derivatives, and their anti-inflammatory activities were explored on LPS-induced inflammatory molecules in RAW 264.7 cells.

The NO is a key signaling molecule and has been well known to regulate various physiological functions in many tissues of the human body [19]. However, an overproduction of NO is associated with many inflammatory diseases, so discovery of natural bioactive compounds plays a key role in research of new drugs for reducing the inflammatory molecules. Therefore, seven new phenylpropanoid compounds were isolated from the methanol extract of the aerial parts of *D. polystachya* by many kinds of chromatographs. The inhibitory rate on LPS-induced expression of nitric oxide in RAW 264.7 cells of all isolated compound were evaluated, and the results showed that compounds (**1**–**3**, **6**) that displayed NO inhibitory activity. Notably, by comparing compound **1** with **3**, we found when the methoxy group located at C-3′ could cause a dramatic reduction in the inhibitory activity. The NO inhibitory activity of compounds **1**–**3** versus **4**–**7** suggested the bibenzyl compounds were more active than benzofuran stilbene compounds. The anti-inflammatory activities of bibenzyl derivatives may due to their special chemical structure, in which a carbon–carbon bond can have free rotation that produces multiple spatial conformations. These results demonstrated that structurally different phenylpropanoid compounds in *D. polystachya* were possible to play their anti-inflammatory function. Furthermore, proinflammatory molecules, including iNOS and COX-2, were involved in inflammation-associated diseases and act as inflammatory mediators or activators of inflammatory pathways, such as NF-κB [20]. Therefore, the expression levels on these proteins were detected by Western blotting. The result show that compound **3** obvious inhibited the expression of iNOS, which proposing compound **3** appears to be mediated NO release via the suppression of NF-κB inflammasome pathway.

In this study, a bibenzyl derivative, diosbiben C (**3**), isolated from the aerial parts of *D. polystachya*, significantly reduced the NO release of LPS-stimulated in RAW 264.7 cells, and the Western blot analysis resulting in a reduction in the expression inflammatory molecules of iNOS in NF-κB pathway. In addition to the NF-κB signaling pathway, our future research will investigate the anti-inflammatory mechanism of diosbiben C in more detail.

## 4. Materials and Methods

### 4.1. General Experimental Procedures

The NMR data were recorded on a Bruker 500 and 600 MHz spectrometer (Bruker, Germany). IR spectra were recorded with KBr disks by a Bruker vertex-70 spectrometer (Bruker, Germany). A high-resolution mass spectrum was acquired via Shimadzu LC-IT-TOF (Shimadzu, Japan). MPLC separation was performed on a Büchi sepacore (Buchi Labortechnik AG, Flawil, Swizerland) with YMC gel ODS C_18_ column (45–60 μm, YMC Co., Ltd., Kyoto, Japan). Column chromatography (CC) was carried out using silica gel (200–300 mesh, Qingdao Marine Chemical Co., Ltd., Qingdao, China) and Sephadex LH-20 (GE Healthcare Bio-Sciences AB, Uppsala, Sweden). Thin-layer chromatography (TLC) was undertaken on HSGF254 plates (Qingdao Marine Chemical Co., Ltd., China). Semi-preparative HPLC was conducted on an LC-3000 semi-preparation gradient HPLC system (Chuangxintongheng, Beijing, China), equipped with a UV–vis detector and a semipreparative RP-HPLC column (Shiseido CAPCELL PAK C_18_ column, 250 × 20 mm, 5 μm, Japan). ACN (HPLC grade) (CINC High Purity Solvents, Shanghai, China). Methanol, acetone, petroleum ether (60–90), ethyl acetate (AR) (Sinopharm Co., Ltd., Shanghai, China), ultrapure water was produced by a Milli-Q water purification system (Milford, MA, USA).

### 4.2. Plant Materials

The fresh aerial parts of *D. polystachya* were collected at Lu’an, Anhui province (China), in September 2019 and identified by associate Prof. Tao Xu.

### 4.3. Extraction and Isolation

The fresh aerial parts of *D. polystachya* (5 kg) were extracted three times with methanol to give a crude extract, then the extract was suspended in water and extracted with EtOAc, affording an EtOAc soluble extract (20 g). The extract was subjected to an MCI gel column CC (46–50 μm) using MeOH-H_2_O (*v/v*) in step gradient from 1:9 to 1:0 to obtain four fractions Fr.1–Fr.4. Fr. 2 (800 mg) was subjected to Sephadex LH-20 column (MeOH) and separated by semipreparative HPLC (MeCN-H_2_O, 50:50 *v/v*) to furnish compound **6** (6.0 mg, *t*_R_ = 24.2 min) and **7** (5.8 mg, *t*_R_ = 29.1 min). Fr. 3 (5 g) was chromatographed through a silica gel column with petroleum ether-acetone (10:1 to 1:1, *v/v*) to give fractions 3-1 to 3-4. Fr. 3-2 (1.1 g) was separated by MPLC eluted with MeOH-H_2_O (20:70–100:0, *v/v*), and followed by Sephadex LH-20 column (MeOH) and purified by pre-HPLC (MeCN-H_2_O, 50:50 *v/v*) to obtain compound **3** (8.0 mg, *t*_R_ = 25.7 min). Fr. 3-3 (2 g) was separated by MPLC eluted with MeOH-H_2_O (20:70–100:0, *v/v*), and purified by pre-HPLC (MeCN-H_2_O, 55:45 *v/v*) to obtain compound **1** (5.0 mg, *t*_R_ = 26.3 min) and **2** (2.6 mg, *t*_R_ = 28.7 min). Fr. 4 (6 g) was chromatographed through MPLC (MeOH-H_2_O, 30:70–100:00, *v/v*) to give five subfractions Fr.4-1–Fr.4-5. Fr.4-3 (100 mg) was chromatographed on Sephadex LH-20 column (eluted with MeOH) and applied to pre-TLC (petroleum ether: acetone 2:1, *v/v*) to yield compound **4** (6.0 mg) and compound **5** (3.0 mg).

Diosbiben A (**1**): yellowish amorphous solid; UV (MeCN) *λ*_max_ 200, 272 nm; IR (KBr) *ν*_max_ 3425, 2938, 1613, 1469, 1517, 1215, 1114, 1000, 791, 752 cm^−1^; ^1^H and ^13^C NMR data (Table 1); HRESIMS *m/z* 303.1238 [M-H]^−^ (calcd for C_17_H_19_O_5_, 303.1238).

Diosbiben B (**2**): yellowish amorphous solid; UV (MeCN) *λ*_max_ 199, 272 nm; ^1^H and ^13^C NMR data (Table 1); HRESIMS *m/z* 303.1238 [M-H]^−^ (calcd for C_17_H_19_O_5_, 303.1238).

Diosbiben C (**3**): yellowish amorphous solid; UV (MeCN) *λ*_max_ 199, 276 nm; IR (KBr) *ν*_max_ 3558, 3290, 2938, 1612, 1518, 1474, 1316, 1274, 1202, 1083, 1000, 804 cm^−1^; ^1^H and ^13^C NMR data (Table 1); HRESIMS *m/z* 303.1238 [M-H]^−^ (calcd for C_17_H_19_O_5_, 303.1238).

Diosbenfura A (**4**): yellow amorphous powder; UV (MeCN) *λ*_max_ 224, 296 nm; IR (KBr) *ν*_max_ 3409, 2940, 2838, 1622, 1515, 1438, 1201, 1150, 1092, 817 cm^−1^; ^1^H and ^13^C NMR data (Table 2 and Table 3); HRESIMS *m/z* 285.0768 [M-H]^-^ (calcd for C_16_H_13_O_5_, 285.0768).

Diosbenfura B (**5**): yellow amorphous powder; UV (MeCN) *λ*_max_ 200, 285 nm; ^1^H and ^13^C NMR data (Table 2 and Table 3); HRESIMS *m/z* 285.0767 [M-H]^−^ (calcd for C16H13O5, 285.0768).

Diosbenfura C (**6**): yellow amorphous powder; UV (MeCN) *λ*_max_ 216, 318 nm; IR (KBr) *ν*_max_ 3428, 2940, 2837, 1618, 1486, 1322, 1209, 1097, 837 cm^−1^; ^1^H and ^13^C NMR data (Table 2 and Table 3); HRESIMS *m/z* 315.0875 [M-H]^−^ (calcd for C_17_H_15_O_6_, 315.0874).

Diosbenfura D (**7**): yellow amorphous powder; UV (MeCN) *λ*_max_ 211, 309 nm; IR (KBr) *ν*_max_ 3414, 2940, 2840, 1605, 1494, 1444, 1348, 1241, 1105, 1030, 770 cm^−1^; ^1^H and ^13^C NMR data (Table 2 and Table 3); HRESIMS *m/z* 285.0767 [M-H]^−^ (calcd for C_16_H_13_O_5_, 285.0768).

### 4.4. Cell Viability Assay

An MTT assay was used to evaluate RAW 264.7 cell viability as previously described. Briefly, cells were plated in 96-well plates (5 × 10^3^ cells/well) for 18 h and then incubated with compounds **1**–**7** in various concentrations with or without LPS (1.0 μg/mL). Eighteen hours later, the prepared MTT solution (20 μL, 5 mg/mL) was added, and the cells were incubated for another 4 h. After the formazan that formed was fully dissolved in DMSO (150 μL/well), the absorbance was read at 570 nm on a microplate reader. The viability of RAW 264.7 cells for the control group (with DMSO only) is defined as 100%.

### 4.5. Cell Culture and NO Production Measurements

The experimental procedures were followed by the literature [21]. Cells were seeded in 96-well plates at the density of 50,000 cells/well for 24 h, pretreated with the tested compounds for 30 min at 37 °C, and co-incubated with LPS (100 ng/mL) for 24 h. NO production was analyzed through Griess reaction. Momently, cell culture supernatant (50 μL) and Griess reagent (50 μL) were mixed for 10 min, and then monitored at 540 nm using a microplate reader. All the tested compounds were prepared as stock solutions with a concentration of 10 mM in DMSO. Aminoguanidine hydrochloride was used as the positive control.

### 4.6. Western Blot Analysis

Cells were pretreated with the test compound **3** for 30 min and stimulated with LPS (1 μg/mL) for 24 h. The total proteins were extracted and immunoblotted as previously described [22,23]. Briefly, the harvested cells were lysed by 1% RIPA (radio-immunoprecipitation assay) (Amresco, Solon, OH, USA) to achieve the cellular lysates. Cellular lysates were centrifuged, and the total protein concentration was measured by the BCA protein assay. Total proteins were electrophoresed on SDS-PAGE and transferred onto a PVDF membrane (Bio-Rad Laboratories, Hercules, CA, USA). The membranes were washed with TBST buffer, blocking with 5% skim milk for 2 h at 25 °C, and then incubated with primary antibodies for 12 h at 4 °C. After being washed with TBST buffer, the membranes were treated with secondary antibody at room temperature.

## 5. Conclusions

In summary, seven undescribed phenylpropanoid derivatives (**1**–**7**) were isolated from the fresh aerial parts of *D. polystachya*. The new compounds were identified as bibenzyl and benzofuran stilbene derivatives. Those compounds will enrich the structural skeletons of natural occurring phenylpropanoids and the structural diversity of the Dioscoreaceae family. All new phenylpropanoid derivatives were screened for anti-inflammatory effects. The screened results revealed that compounds (**1**–**3**, **6**) displayed strong NO inhibitory activity with the IC_50_ range of 9.3–32.3 μM. Importantly, the potential compound **3** decreased iNOS levels, and indicated that **3** may mediated via the suppression of an LPS-induced NF-κB inflammasome pathway. These findings provide an insight into the potential therapeutic value of phenylpropanoid derivatives for inflammatory diseases. However, more studies are needed to determine whether these compounds can act on other inflammatory mechanisms.

## Figures and Tables

**Table 1:** ^1^H NMR and ^13^C NMR spectroscopic data of **1**–**3** in CD_3_OD (*δ* in ppm, *J* in Hz).

Position	1 ^a^		2 ^b^		3 ^a^	
	*δ*_H_ (*J* in Hz)	*δ*_C_	*δ*_H_ (*J* in Hz)	*δ*_C_	*δ*_H_ (*J* in Hz)	*δ*_C_
1	-	134.1, C	-	139.5, C	-	134.2, C
2	6.37 (s)	106.7, CH	6.33 (d, 1.6)	110.1, CH	6.30 (d, 1.4)	109.9, CH
3	-	149.0, C	-	151.2, C	-	146.3, C
4	-	134.5, C	-	135.8, C	-	133.1, C
5	-	149.0, C	-	154.2, C	-	149.4, C
6	6.37 (s)	106.7, CH	6.27 (d, 1.6)	105.2, CH	6.23 (d, 1.4)	104.9, C
1″	2.78 (m)	38.1, CH_2_	2.73 (m)	38.2, CH_2_	2.69 (m)	38.1, CH_2_
2″	2.84 (m)	33.2, CH_2_	2.83 (m)	33.1, CH_2_	2.82 (m)	33.3, CH_2_
1′	-	136.6, C	-	136.6, C	-	136.7, C
2′	-	147.2, C	-	147.2, C	-	148.3, C
3′	-	151.0, C	-	151.2, C	-	154.0, C
4′	6.67 (dd, 7.8, 1.4)	115.4, CH	6.67 (dd, 7.7, 1.2)	115.5, CH	6.80 (d, 7.8)	111.6, CH
5′	6.80 (t, 7.8)	125.0, CH	6.81 (t, 7.7)	125.1, CH	6.92 (t, 7.8)	124.9, CH
6′	6.59 (dd, 7.8, 1.4)	122.1, CH	6.62 (dd, 7.7, 1.2)	121.9, CH	6.70 (d, 7.8)	123.2, CH
3-OCH_3_	3.75 (s)	56.6, CH_3_	-	-	-	-
4-OCH_3_	-	-	3.72 (s)	60.9, CH_3_	-	-
5-OCH_3_	3.75 (s)	56.6, CH_3_	3.75 (s)	56.2, CH_3_	3.71 (s)	61.0, CH_3_
2′-OCH_3_	3.70 (s)	60.8, CH_3_	3.72 (s)	60.9, CH_3_	3.71 (s)	61.0, CH3
3′-OCH_3_	-	-	-	-	3.79 (s)	56.1, CH_3_

^a^: ^1^H NMR was recorded in 500 MHz and ^13^C NMR was recorded in 125 MHz; ^b^: ^1^H NMR was recorded in 600 MHz and ^13^C NMR was recorded in 150 MHz.

**Table 2:** ^1^H NMR spectroscopic data of **4**–**7** in CD_3_OD (*δ* in ppm, *J* in Hz).

Position	*δ*_H_ (*J* in Hz)			
	4 ^a^	5 ^b^	6 ^a^	7 ^a^
2	7.38 (d, 8.5)	7.04 (d, 2.5)	7.06 (s)	6.58 (d, 7.8)
3	6.82 (dd, 8.5, 2.0)	-	-	7.06 (t, 7.8)
4	-	6.81 (dd, 8.8, 2.5)	-	6.98 (d, 7.8)
5	7.07 (d, 2.0)	7.33 (d, 8.8)	7.14 (s)	-
1″	6.85 (s)	6.89 (s)	6.83 (s)	7.07 (s)
2′	6.96 (s)	6.98 (d, 1.8)	6.95 (s)	6.99 (s)
6′	6.96 (s)	6.99 (d, 1.8)	6.95 (s)	6.99 (s)
3-OCH_3_	-	3.81 (s)	3.85 (s)	-
4-OCH_3_	3.83 (s)	-	3.87 (s)	-
3′-OCH_3_	3.91 (s)	3.92 (s)	3.91 (s)	3.91 (s)
4′-OCH_3_	-	-	-	3.81 (s)

^a^: ^1^H NMR was recorded in 500 MHz; ^b^: ^1^H NMR was recorded in 600 MHz.

**Table 3:** ^13^C NMR spectroscopic data of **4**–**7** in CD_3_OD (*δ* in ppm).

Position	*δ*_C_			
	4 ^a^	5 ^b^	6 ^a^	7 ^a^
1	124.2, C	131.5, C	123.2, C	119.8, C
2	121.6, CH	104.1, CH	104.0, CH	108.7, CH
3	112.6, CH	157.6, C	147.9, C	126.0, CH
4	159.2, C	113.2, CH	149.1, C	103.6, CH
5	96.6, CH	112.0, CH	100.7, CH	152.0, C
6	156.9, C	150.9, C	150.7, C	152.1, C
1″	100.3, CH	100.7, CH	96.6, CH	99.2, CH
2″	156.9, C	158.6, C	157.0, C	157.7, C
1′	123.1, C	122.8, C	123.1, C	127.7, C
2′	106.5, CH	106.8, CH	106.4, CH	106.6, CH
3′	149.9, C	149.9, C	149.8, C	154.9, C
4′	135.9, C	136.4, C	135.8, C	138.1, C
5′	146.8, C	146.9, C	146.8, C	155.3, C
6′	101.2, CH	101.5, CH	101.1, CH	101.4, CH
3-OCH_3_	-	56.2, CH_3_	57.0, CH_3_	-
4-OCH_3_	56.2, CH_3_	-	56.8, CH_3_	-
3′-OCH_3_	56.7, CH_3_	56.7, CH_3_	56.6, CH_3_	56.5, CH_3_
4′-OCH_3_	-	-	-	61.0, CH_3_

^a^: ^13^C NMR was recorded in 125 MHz; ^b^: ^13^C NMR was recorded in 150 MHz.

**Table 4:** IC_50_ values of compounds **1**–**7** inhibiting NO production in RAW 246.7 cells.

Compound	IC_50_ (μM) ^a^
**1**	32.3 ± 0.82
**2**	28.6 ± 1.41
**3**	9.3 ± 1.03
**4**	>50
**5**	>50
**6**	24.1 ± 1.21
**7**	>50
AH ^b^	19.2 ± 0.78

^a^: IC_50_ values were expressed as mean ± SD of three independent experiments. ^b^: AH = Aminoguanidine hydrochloride was used as the positive control.

## Data Availability

The data and materials used in the current study are available from supplementary materials.

## Supplementary Materials

The supporting information can be downloaded at: https://www.mdpi.com/article/10.3390/ijms231810954/s1.

## References

[1] Lim J.S., Oh J., Yun H.S., Lee J.S., Hahn D., Kim J.S. (2022). Anti-neuroinflammatory activity of 6,7-dihydroxy-2,4-dimethoxy phenanthrene isolated from *Dioscorea batatas* decne partly through suppressing the p38 MAPK/NF-kappa B pathway in BV2 microglial cells. J. Ethnopharmacol..

[2] Adomeniene A., Venskutonis P.R. (2022). *Dioscorea* spp.: Comprehensive review of antioxidant properties and their relation to phytochemicals and health benefits. Molecules.

[3] Sautour M., Mitaine-Offer A.C., Lacaille-Dubois M.A. (2007). The *Dioscorea* genus: A review of bioactive steroid saponins. J. Nat. Med..

[4] Fan Y.J., He Q.Y., Luo A.S., Wang M.Y., Luo A.X. (2015). Characterization and antihyperglycemic activity of a polysaccharide from *Dioscorea opposita* Thunb roots. Int. J. Mol. Sci..

[5] Koo H.J., Park H.J., Byeon H.E., Kwak J.H., Um S.H., Kwon S.T., Rhee D.K., Pyo S. (2014). Chinese yam extracts containing beta-sitosterol and ethyl linoleate protect against atherosclerosis in apolipoprotein E-deficient mice and inhibit muscular expression of VCAM-1 in vitro. J. Food Sci..

[6] Liu Y.H., Lin Y.S., Liu D.Z., Han C.H., Chen C.T., Fan M., Hou W.C. (2009). Effects of different types of *Yam* (*Dioscorea alata*) products on the blood pressure of spontaneously hypertensive rats. Biosci. Biotechnol. Biochem..

[7] Byeon S., Oh J., Lim J.S., Lee J.S., Kim J.S. (2018). Protective effects of *Dioscorea batatas* flesh and peel extracts against ethanol-induced gastric ulcer in mice. Nutrients.

[8] Koo H.J., Lee S., Chang K.J., Sohn E., Sohn E.H., Kang S.C., Pyo S. (2017). Hepatic anti-inflammatory effect of hexane extracts of *Dioscorea batatas* decne: Possible suppression of toll-like receptor 4-mediated signaling. Biomed. Pharmacother..

[9] Go H.K., Rahman M.M., Kim G.B., Na C.S., Song C.H., Kim J.S., Kim S.J., Kang H.S. (2015). Antidiabetic effects of *Yam* (*Dioscorea batatas*) and its active constituent, allantoin, in a rat model of streptozotocin-induced diabetes. Nutrients.

[10] Gugu F.S., Paul S., Simon G. (2020). Constituents of two *Dioscorea* species that potentiate antibiotic activity against MRSA. J. Nat. Prod..

[11] Ma C., Wang W., Chen Y.Y., Liu R.N., Wang R.F., Du L.J. (2005). Neuroprotective and antioxidant activity of compounds from the aerial parts of *Dioscorea opposite*. J. Nat. Prod..

[12] Dong S.H., Nikolic D., Simmler C., Qu F., van Breemen R.B., Soejarto D.D., Pauli G.F., Chen S.N. (2013). Diarylheptanoids from *Dioscorea villosa* (Wild Yam). J. Nat. Prod..

[13] Wang J.T., Ge D., Qu H.F., Wang G.K., Wang G. (2019). Chemical constituents of *Curcuma kwangsiensis* and their antimigratory activities in RKO cells. Nat. Prod. Res..

[14] Wang J.T., Zhang P.L., Liu J.S., Wang G.K., Xu F.Q., Chen L., Yu Y., Wang G. (2018). Aspergilates A to E, second metabolites from Aspergillus sp. isolated from Paeonia ostia. Fitoterapia.

[15] Yue J.Y., Wang R., Xu T., Wang J.T., Yu Y., Cai B.X. (2022). Novel phenolic metabolites isolated from plant endophytic fungus *Fusarium guttiforme*. Nat. Prod. Res..

[16] Zhou X.M., Zheng C.J., Gan L.S., Chen G.Y., Zhang X.P., Song X.P., Li G.N., Sun C.G. (2016). Bioactive phenanthrene and bibenzyl derivatives from the stems of *Dendrobium nobile*. J. Nat. Prod..

[17] Wang W.J., Wang L., Liu Z., Jiang R.W., Liu Z.W., Li M.M., Zhang Q.W., Dai Y., Li Y.L., Zhang X.Q. (2016). Antiviral benzofurans from *Eupatorium chinense*. Phytochemistry.

[18] Hong C.J., Chen S.Y., Hsu Y.H., Yen G.C. (2022). Protective effect of fermented okara on the regulation of inflammation, the gut microbiota, and SCFAs production in rats with TNBS-induced colitis. Food Res. Int..

[19] Smith T.L., Oubaha M., Cagnone G., Boscher C., Kim J.S., El Bakkouri Y., Zhang Y., Chidiac R., Corriveau J., Delisle C. (2022). eNOS controls angiogenic sprouting and retinal neovascularization through the regulation of endothelial cell polarity. Cell. Mol. Life Sci..

[20] Erkoc P., Schmitt M., Ingelfinger R., Bischoff-Kont I., Kopp L., Bode H.B., Schiffmann S., Furst R. (2021). Xenocoumacin 2 reduces protein biosynthesis and inhibits inflammatory and angiogenesis-related processes in endothelial cells. Biomed. Pharmacother..

[21] Cai B.X., Song L.X., Hu H.J., Han Z.Z., Zhou Y., Wang Z.T., Yang L. (2021). Structures and biological evaluation of phenylpropanoid derivatives from *Dendrobium Sonia*. Nat. Prod. Res..

[22] Chen J.N., de Mejia E.G., Wu J.S.B. (2011). Inhibitory effect of a glycoprotein isolated from golden oyster mushroom (*Pleurotus citrinopileatus*) on the lipopolysaccharide-induced inflammatory reaction in RAW 264.7 Macrophage. J. Agric. Food Chem..

[23] Hankittichai P., Buacheen P., Pitchakarn P., Takuathung M.N., Wikan N., Smith D.R., Potikanond S., Nimlamool W. (2020). *Artocarpus lakoocha* extract inhibits LPS-induced inflammatory response in RAW 264.7 macrophage cells. Int. J. Mol. Sci..

