# Supplementary material for: Structures and Anti-Inflammatory Evaluation of Phenylpropanoid Derivatives from the Aerial Parts of Dioscorea polystachya
Source: Int J Mol Sci. 2022 Sep 19;23(18):10954. doi: 10.3390/ijms231810954 (PMC9502174; doi:10.3390/ijms231810954)
Supplement: Supplementary file 1 [file ijms-23-10954-s001.zip › ijms-1925904-supplementary.pdf]

Supplementary information

Structures and Anti-Inflammatory Evaluation of Phenylpropanoid Derivatives from the Aerial Parts of *Dioscorea polystachya*

Baixiang Cai [1,2,†], Xinyin Cai [3,4,†], Tao Xu [5], Jutao Wang [1,2,6,*] and Yang Yu [1,7,*]

1 School of Pharmacy, Anhui University of Chinese Medicine, Hefei 230012, China
2 Institute of Medicinal Chemistry, Anhui Academy of Chinese Medicine, Hefei 230012, China
3 The MOE Key Laboratory of Standardization of Chinese Medicines and the SATCM Key Laboratory of New Resources and Quality Evaluation of Chinese Medicines, Shanghai University of Traditional Chinese Medicine, Shanghai 201203, China
4 Institute of Chinese Materia Medica, Shanghai University of Traditional Chinese Medicine, Shanghai 201203, China
5 Department of Biological and Pharmaceutical Engineering, West Anhui University, Luan 237012, China
6 Anhui Province Key Laboratory of Research & Development of Chinese Medicine, Hefei 230012, China
7 Institute for Pharmacodynamics and Safety Evaluation of Chinese Medicine, Anhui Academy of Chinese Medicine, Hefei 230012, China
* Correspondence: wjt591@ahtcm.edu.cn (J.W.); ywhxyu@126.com or yuyang1@mail.kib.ac.cn (Y.Y.)
† These authors contributed equally to this work.

Contents of supplemental material

Figure S1-1-1-6. NMR spectra of compound **1**.

Figure S1-7. (-)-HRESIMS of compound **1**.

Figure S1-8. UV of compound **1**.

Figure S1-9. IR of compound **1**.

Figure S2-1-2-6. NMR spectra of compound **2**.

Figure S2-7. (-)-HRESIMS of compound **2**.

Figure S2-8. UV of compound **2**.

Figure S3-1-3-6. NMR spectra of compound **3**.

Figure S3-7. (-)-HRESIMS of compound **3**.

Figure S3-8. UV of compound **3**.

Figure S3-9. IR of compound **3**.

Figure S4-1-4-6. NMR spectra of compound **4**.

Figure S4-7. (-)-HRESIMS of compound **4**.

Figure S4-8. UV of compound **4**.

Figure S4-9. IR of compound **4**.

Figure S5-1-5-6. NMR spectra of compound **5**.

Figure S5-7. (-)-HRESIMS of compound **5**.

Figure S5-8. UV of compound **5**.

Figure S6-1-6-6. NMR spectra of compound **6**.

Figure S6-7. (-)-HRESIMS of compound **6**.

Figure S6-8. UV of compound **6**.

Figure S6-9. IR spectra of compound **6**.

Figure S7-1-7-6. NMR spectra of compound **7**.

Figure S7-7. (-)-HRESIMS of compound **7**.

Figure S7-8. UV of compound **7**.

Figure S7-9. IR of compound **7**.

Figure S8. ROESY correlations of compounds **1**-**7**.

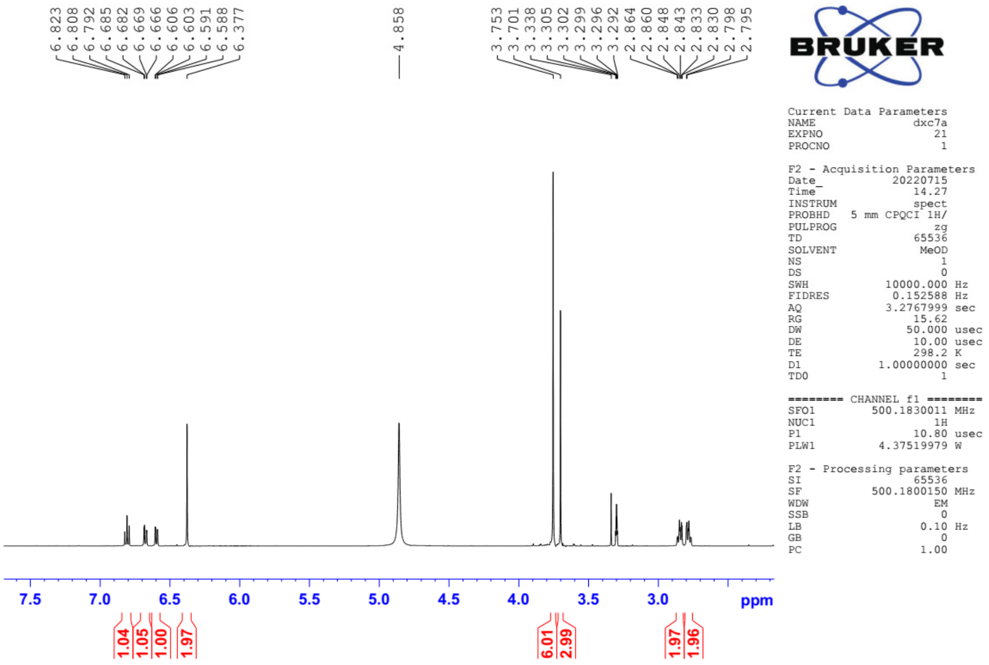

Figure S1-1. ^{1}H NMR (500 MHz, CD_3OD) spectrum of compound **1**.

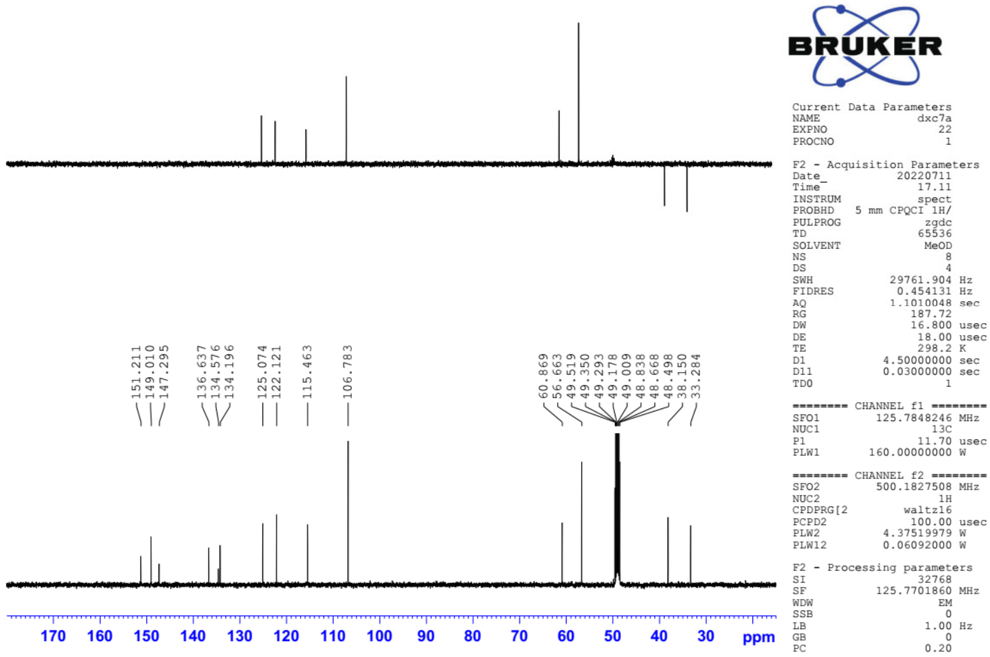

Figure S1-2. ^{13}C NMR and DEPT (125MHz, CD_3OD) spectrum of compound **1**.

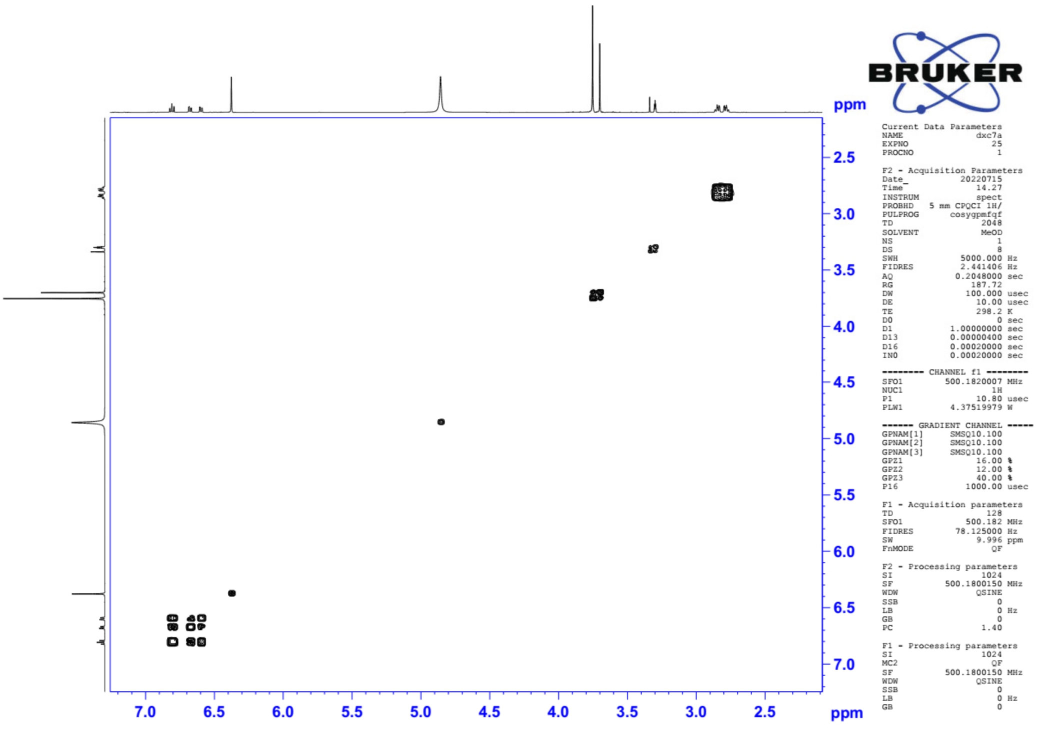

Figure S1-3. ^{1}H-^{1}H COSY spectrum of compound **1**.

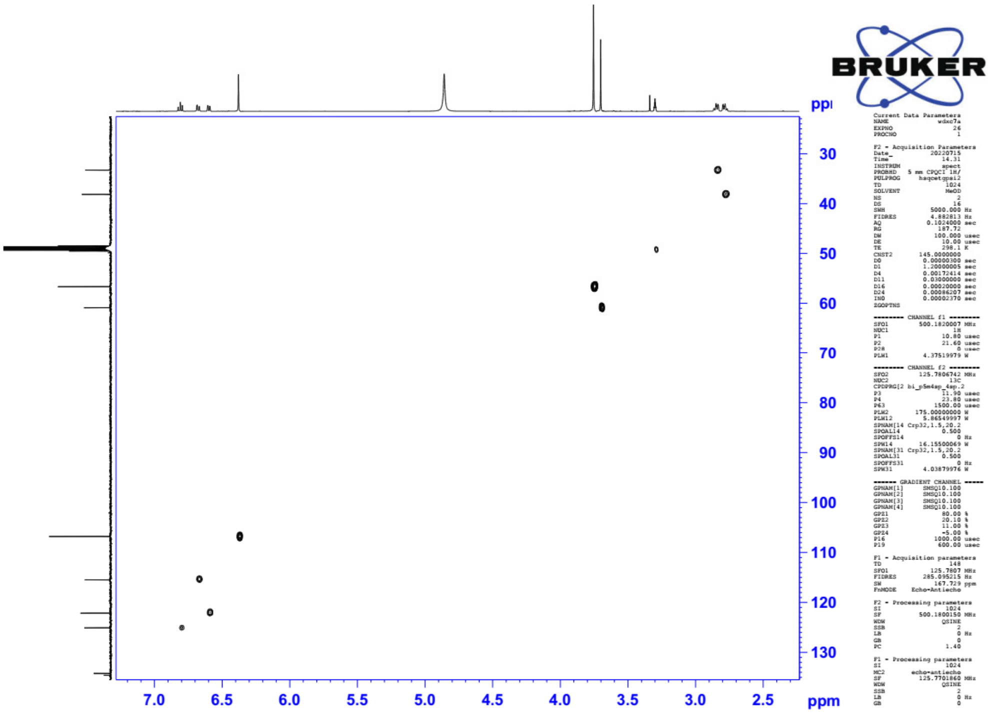

Figure S1-4. HSQC spectrum of compound **1**.

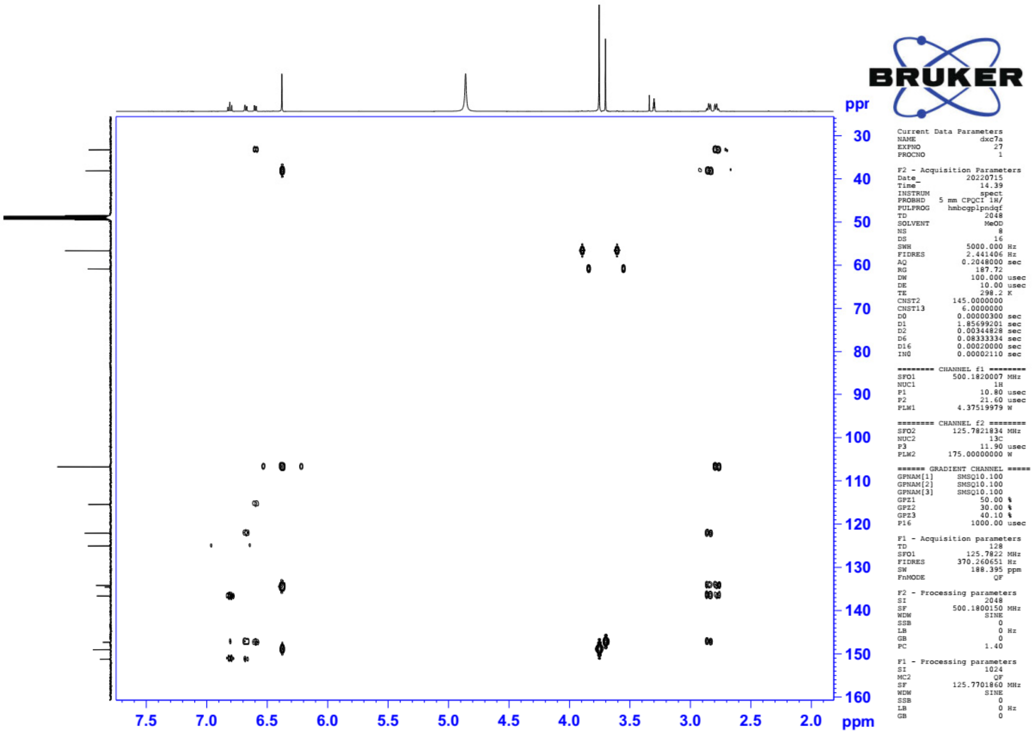

Figure S1-5. HMBC spectrum of compound **1**.

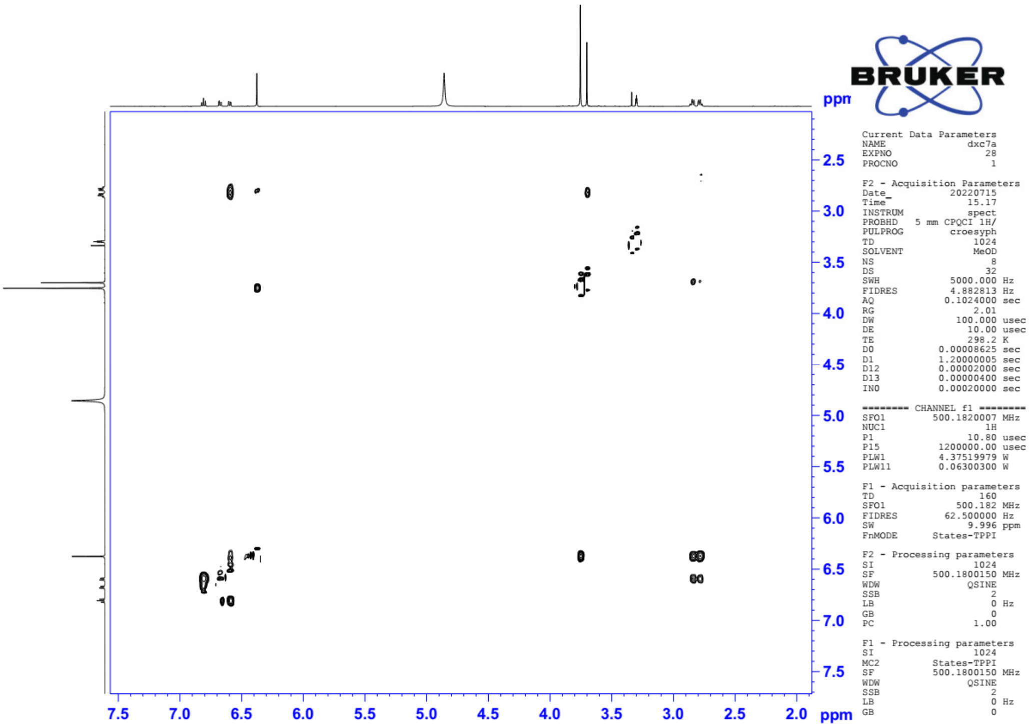

Figure S1-6. ROESY spectrum of compound **1**.

Error Margin (ppm):	5	DBE Range:	not fixed	Electron Ions:	both
HC Ratio:	unlimited	Apply N Rule:	no	Use MSn Info:	yes
Max Isotopes:	all	Isotope RI (%):	1.00	Isotope Res:	10000
MSn Iso RI (%):	75.00	MSn Logic Mode:	OR	Max Results:	30

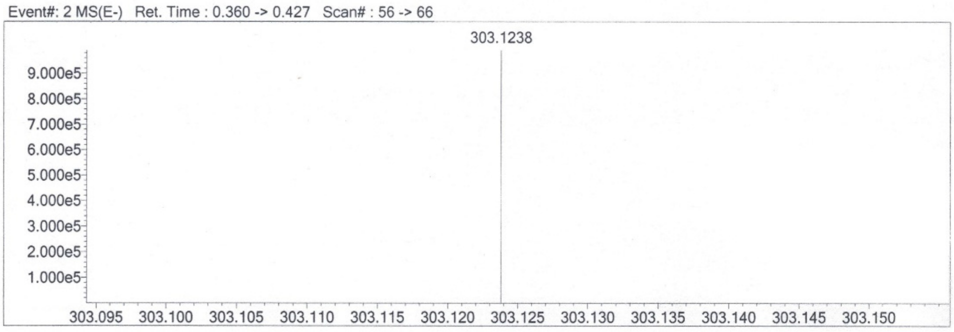

Formula (M)	Ion	Meas. m/z	Pred. m/z	Df. (mDa)	Df. (ppm)	DBE
C17 H20 O5	[M-H]-	303.1238	303.1238	0.0	0.00	8.0

Figure S1-7. (-)-HRESIMS of compound **1**.

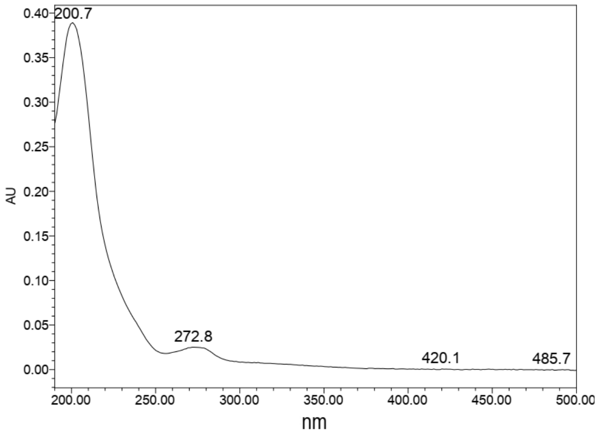

Figure S1-8. UV spectra of compound **1**.

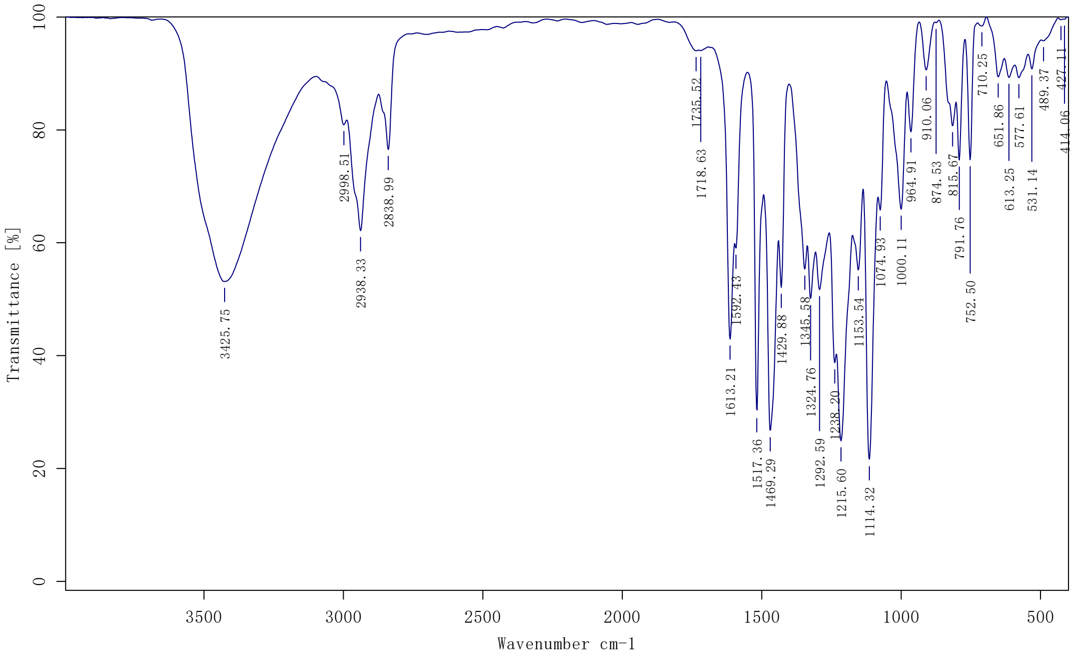

Figure S1-9. IR spectra of compound **1**.

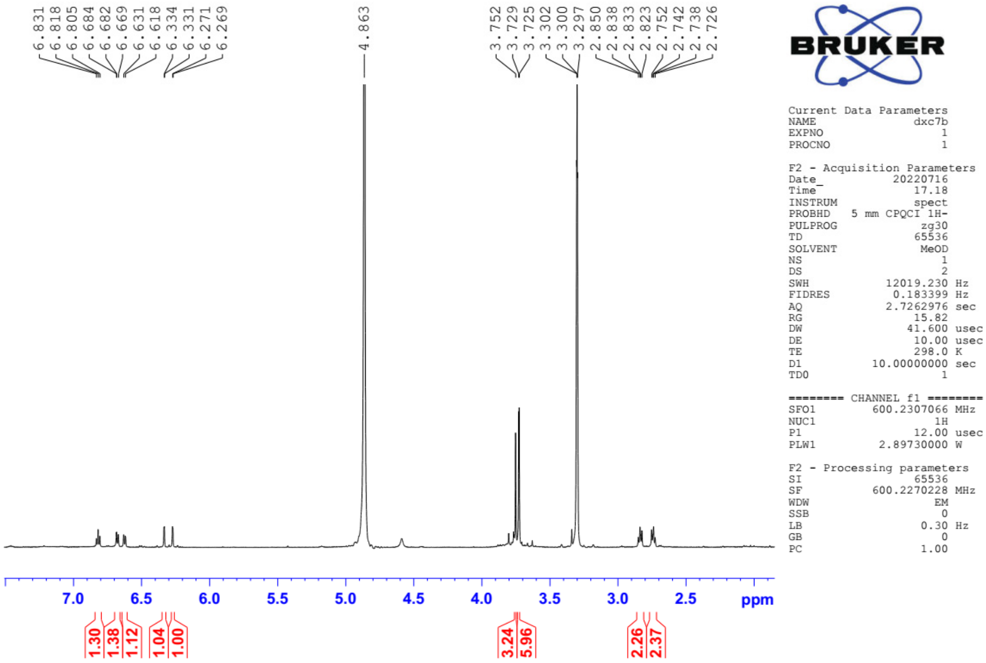

Figure S2-1. ^{1}H NMR (600 MHz, CD_3OD) spectrum of compound **2**.

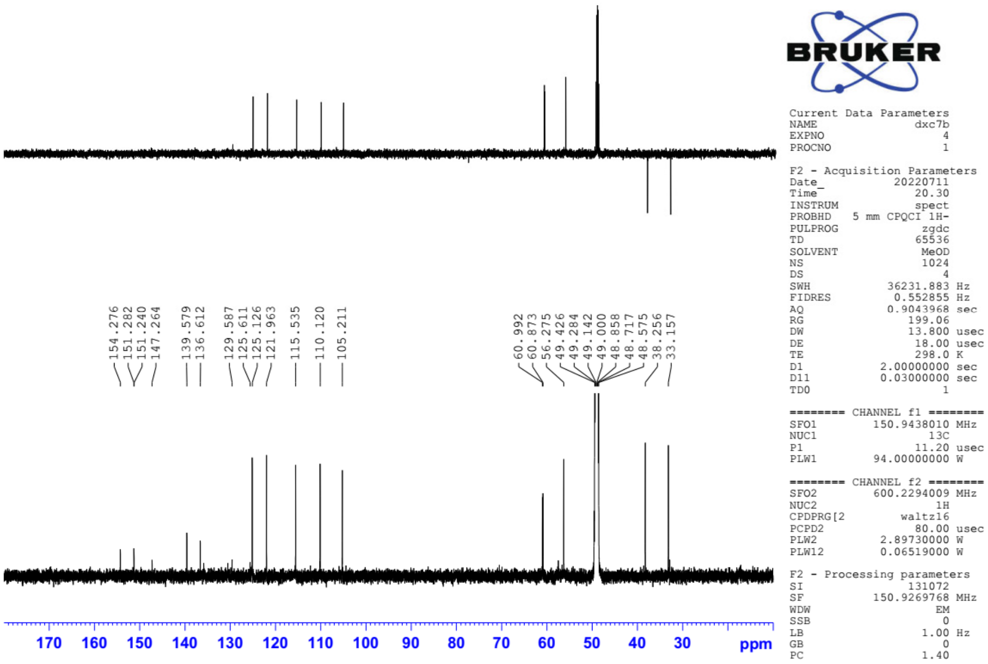

Figure S2-2. ^{13}C NMR and DEPT (150 MHz, CD_3OD) spectrum of compound **2**.

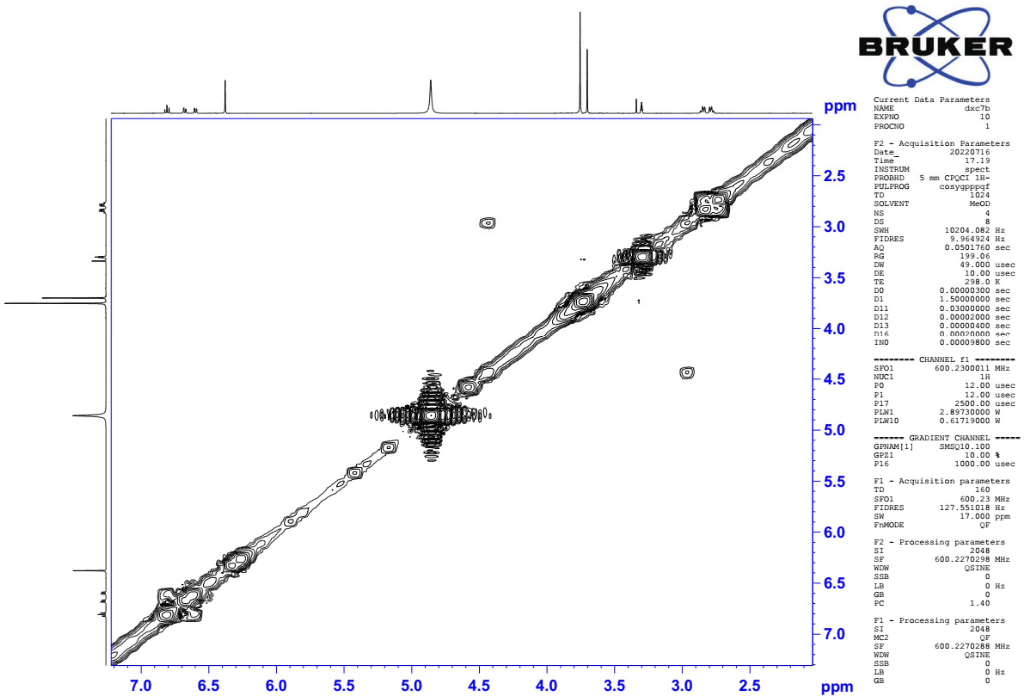

Figure S2-3. ^{1}H-^{1}H COSY spectrum of compound **2**.

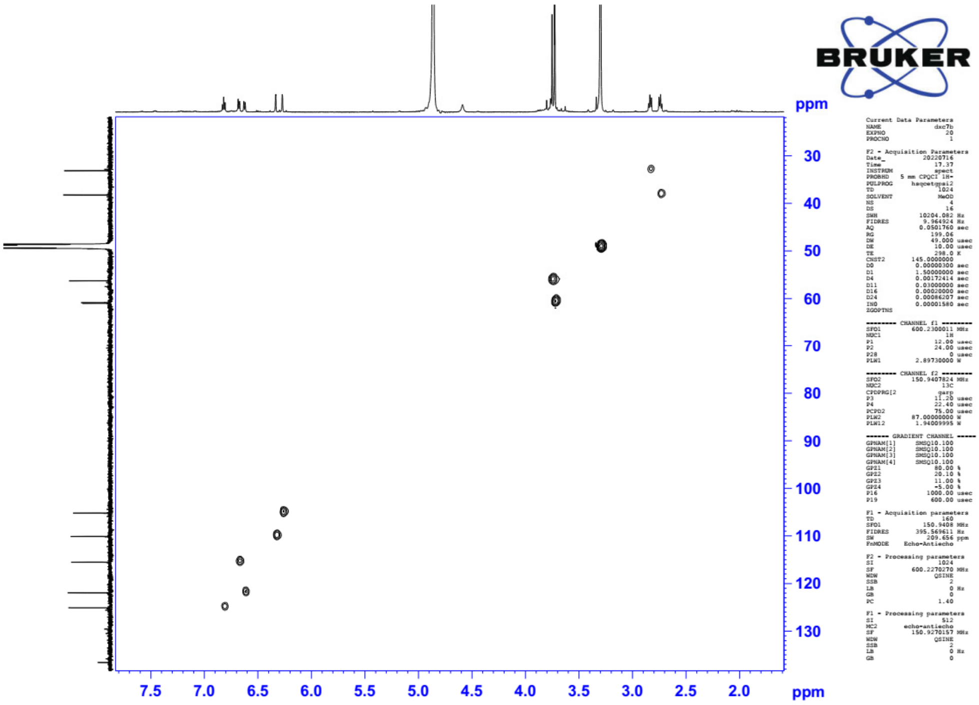

Figure S2-4. HSQC spectrum of compound **2**.

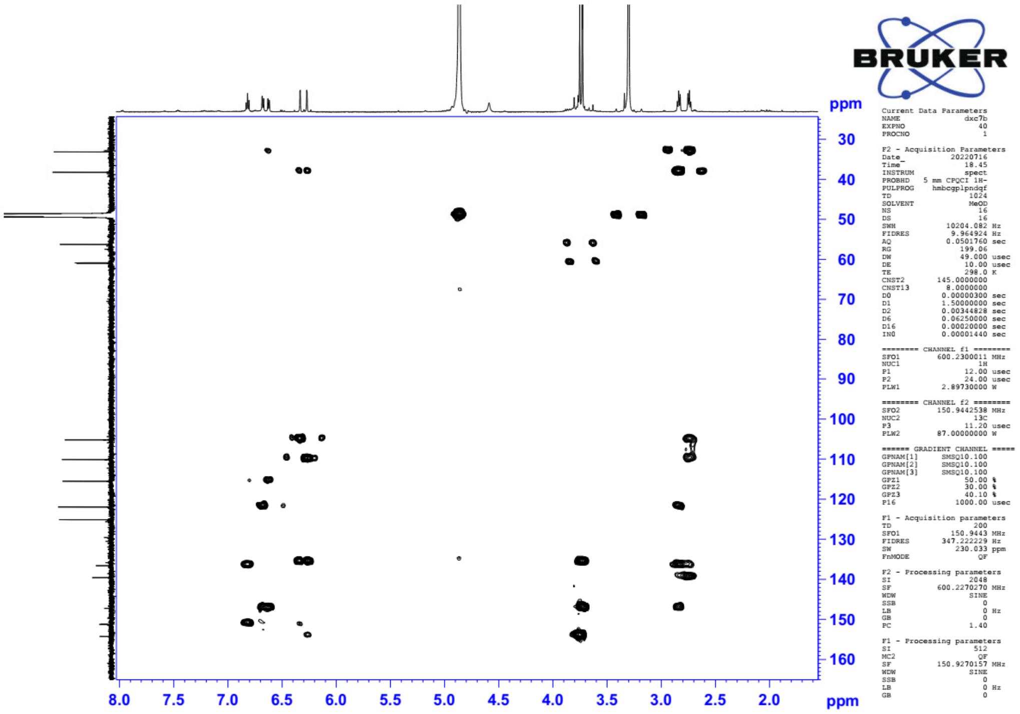

Figure S2-5. HMBC spectrum of compound **2**.

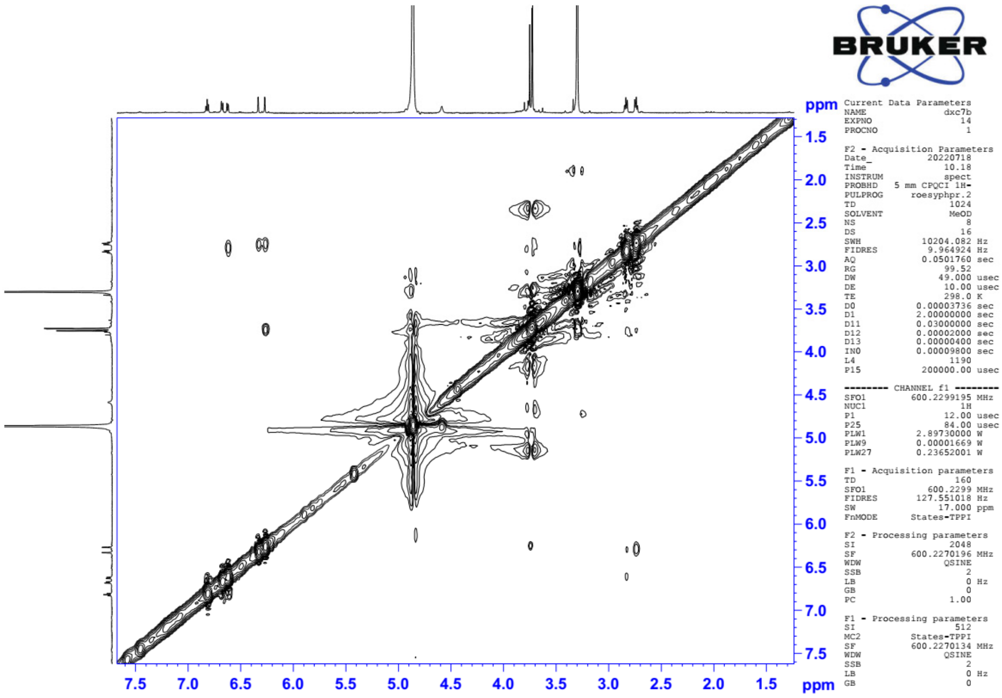

Figure S2-6. ROESY spectrum of compound **2**.

Error Margin (ppm):	5	DBE Range:	not fixed	Electron Ions:	both
HC Ratio:	unlimited	Apply N Rule:	no	Use MSn Info:	yes
Max Isotopes:	all	Isotope RI (%):	1.00	Isotope Res:	10000
MSn Iso RI (%):	75.00	MSn Logic Mode:	OR	Max Results:	30

Event#: 2 MS(E-) Ret. Time : 0.333 -> 0.573 Scan# : 52 -> 88

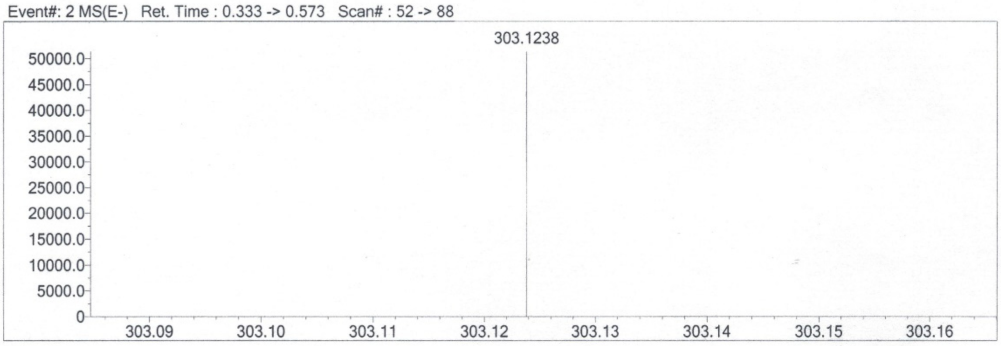

Formula (M)	Ion	Meas. m/z	Pred. m/z	Df. (mDa)	Df. (ppm)	DBE
C17 H20 O5	[M-H]-	303.1238	303.1238	0.0	0.00	8.0

Figure S2-7. (-)-HRESIMS of compound **2**.

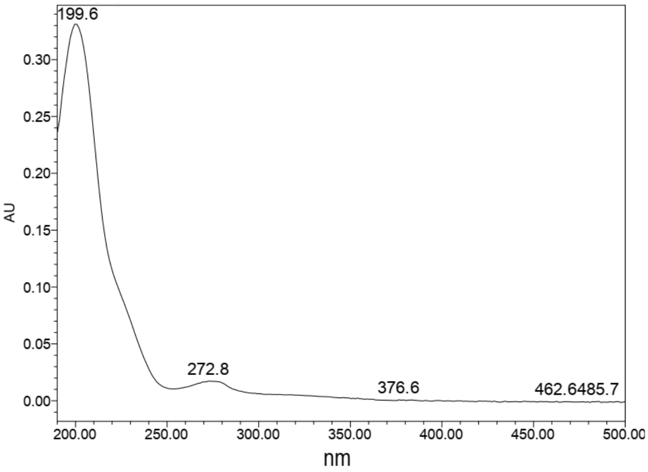

Figure S2-8. UV of compound **2**.

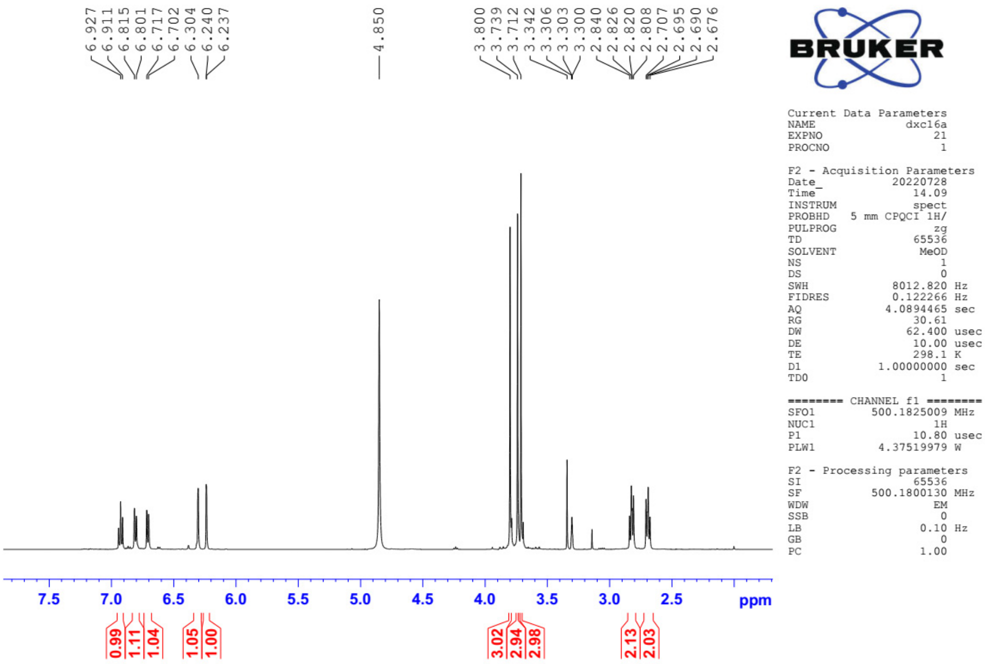

Figure S3-1. ^{1}H NMR (500 MHz, CD_3OD) spectrum of compound **3**.

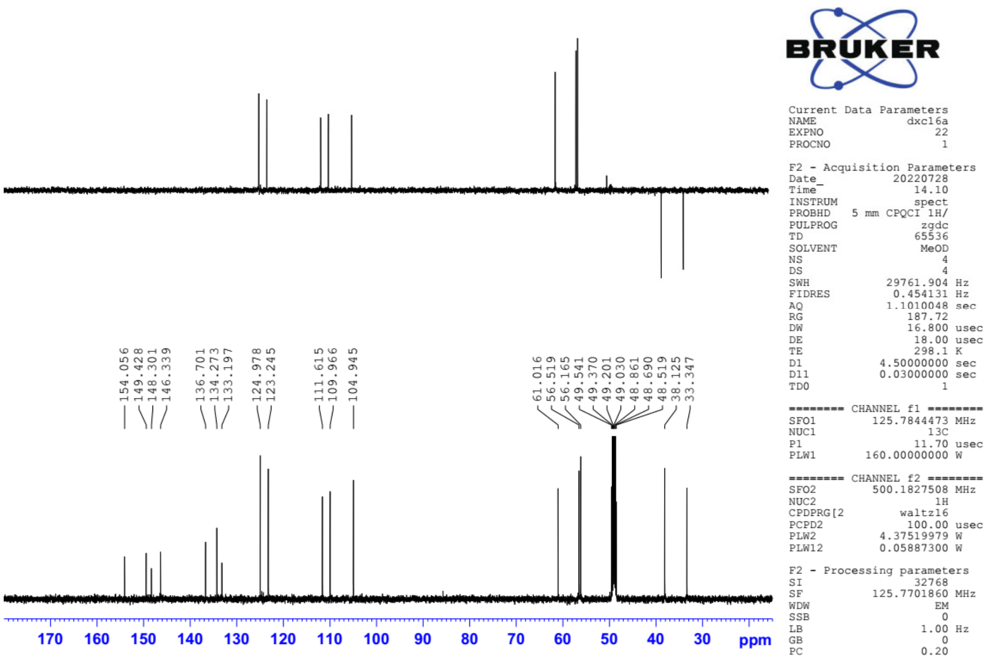

Figure S3-2. ^{13}C NMR and DEPT (125 MHz, CD_3OD) spectrum of compound **3**.

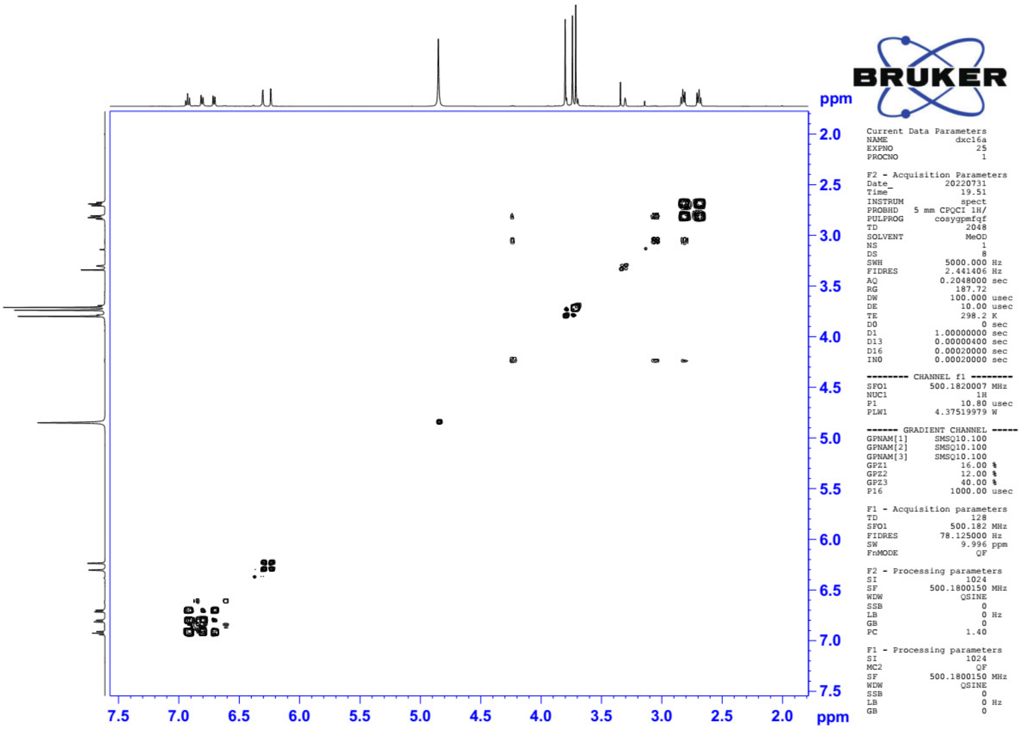

Figure S3-3. ^{1}H-^{1}H COSY spectrum of compound **3**.

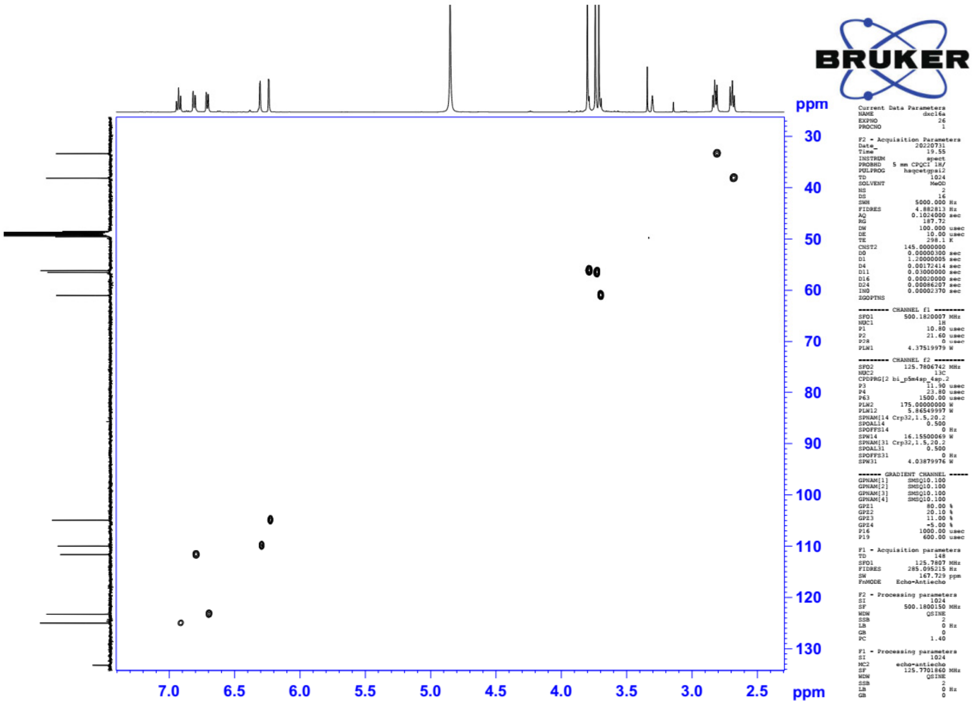

Figure S3-4. HSQC spectrum of compound **3**.

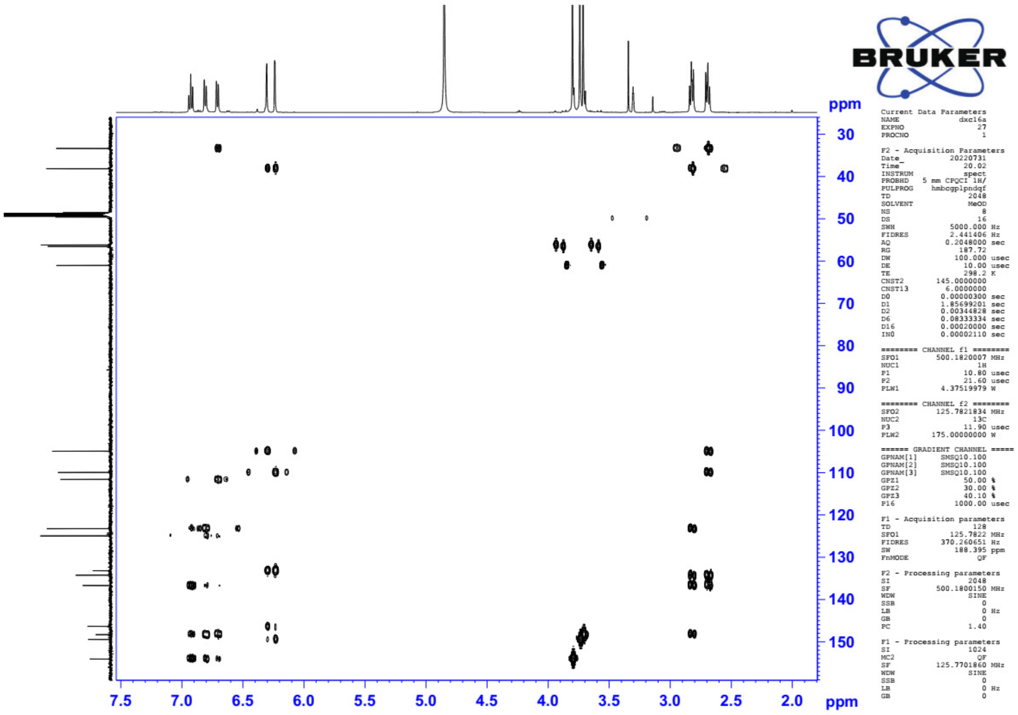

Figure S3-5. HMBC spectrum of compound **3**.

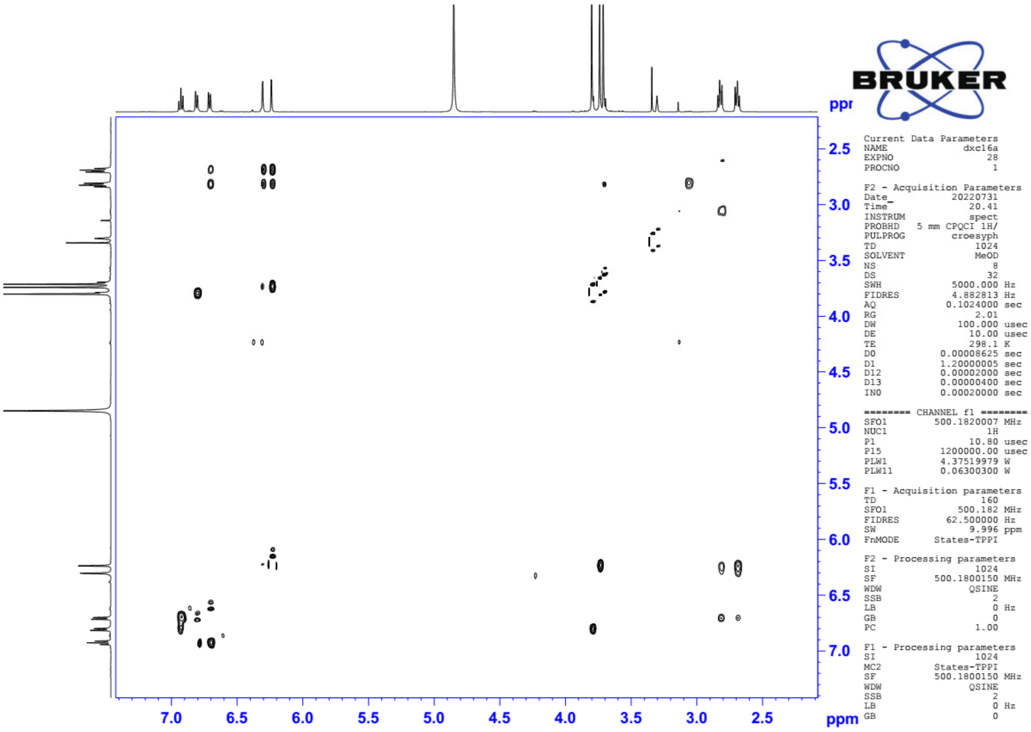

Figure S3-6. ROESY spectrum of compound **3**.

Error Margin (ppm):	5	DBE Range:	not fixed	Electron Ions:	both
HC Ratio:	unlimited	Apply N Rule:	no	Use MSn Info:	yes
Max Isotopes:	all	Isotope RI (%):	1.00	Isotope Res:	10000
MSn Iso RI (%):	75.00	MSn Logic Mode:	OR	Max Results:	30

Event#: 2 MS(E-) Ret. Time : 0.587 -> 0.613 Scan# : 90 -> 94

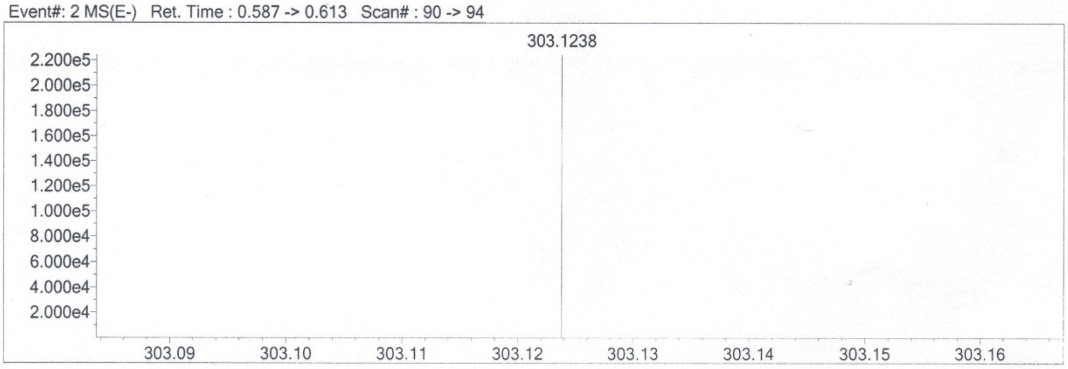

Formula (M)	Ion	Meas. m/z	Pred. m/z	Df. (mDa)	Df. (ppm)	DBE
C17 H20 O5	[M-H]-	303.1238	303.1238	0.0	0.00	8.0

Figure S3-7. (-)-HRESIMS of compound **3**.

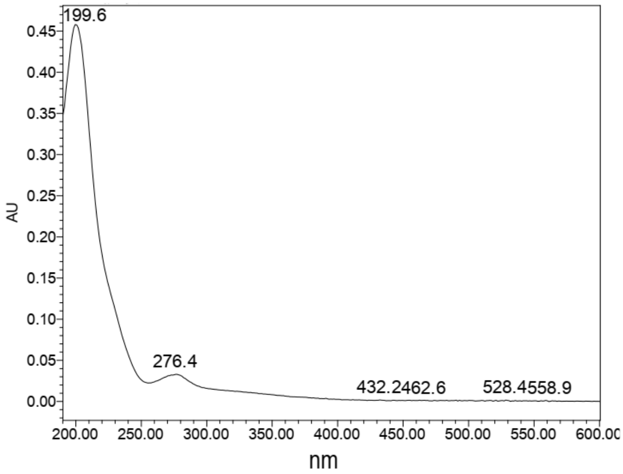

Figure S3-8. UV spectra of compound **3**.

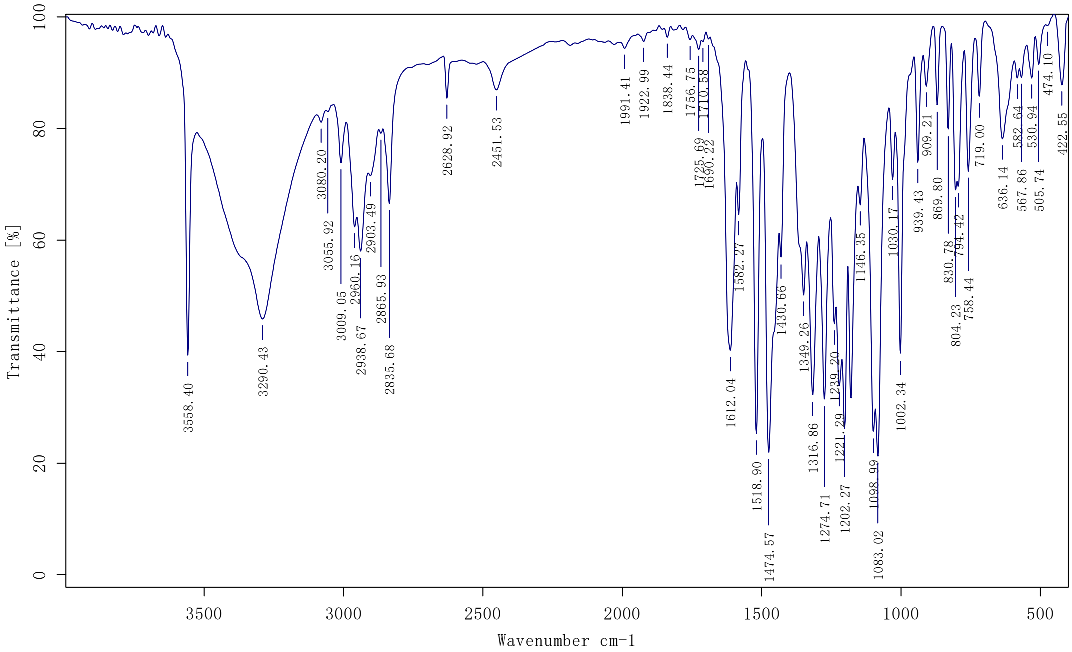

Figure S3-9. IR spectra of compound **3**.

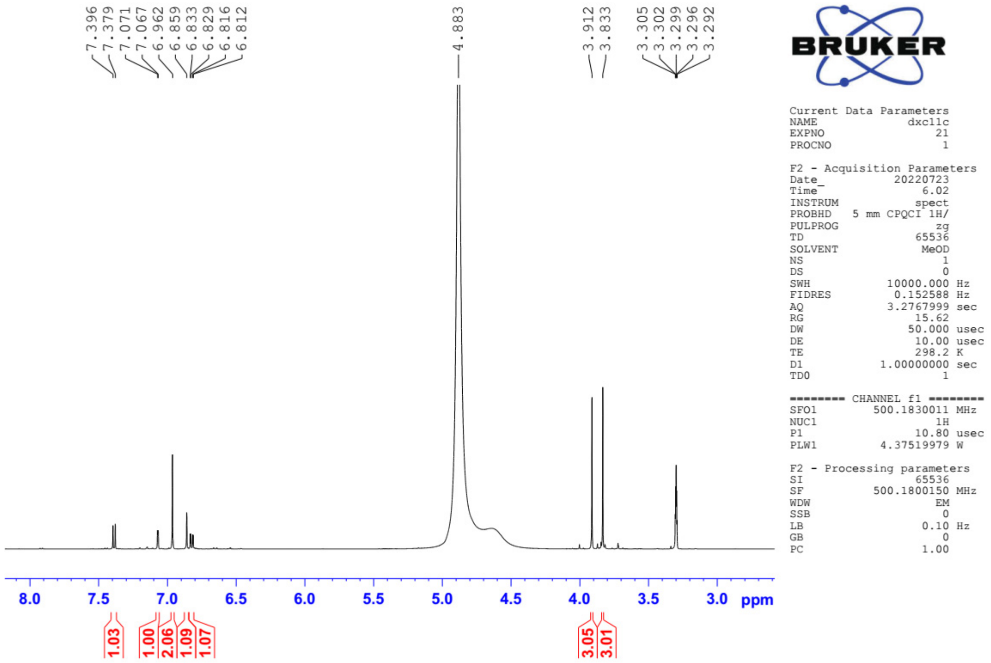

Figure S4-1. ^{1}H NMR (500 MHz, CD_3OD) spectrum of compound **4**.

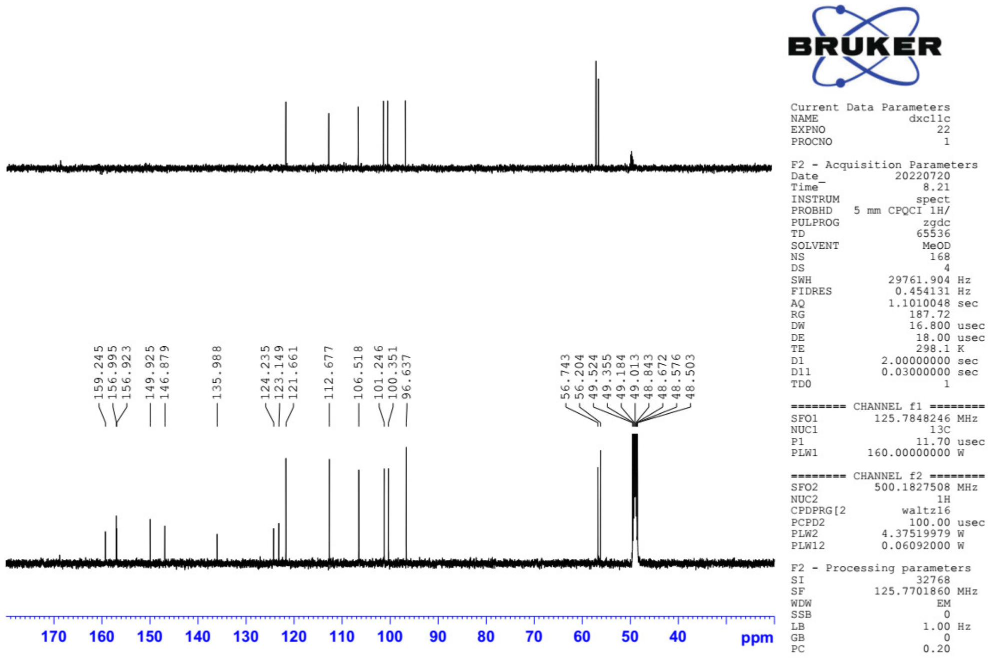

Figure S4-2. ^{13}C NMR and DEPT (125 MHz, CD_3OD) spectrum of compound **4**.

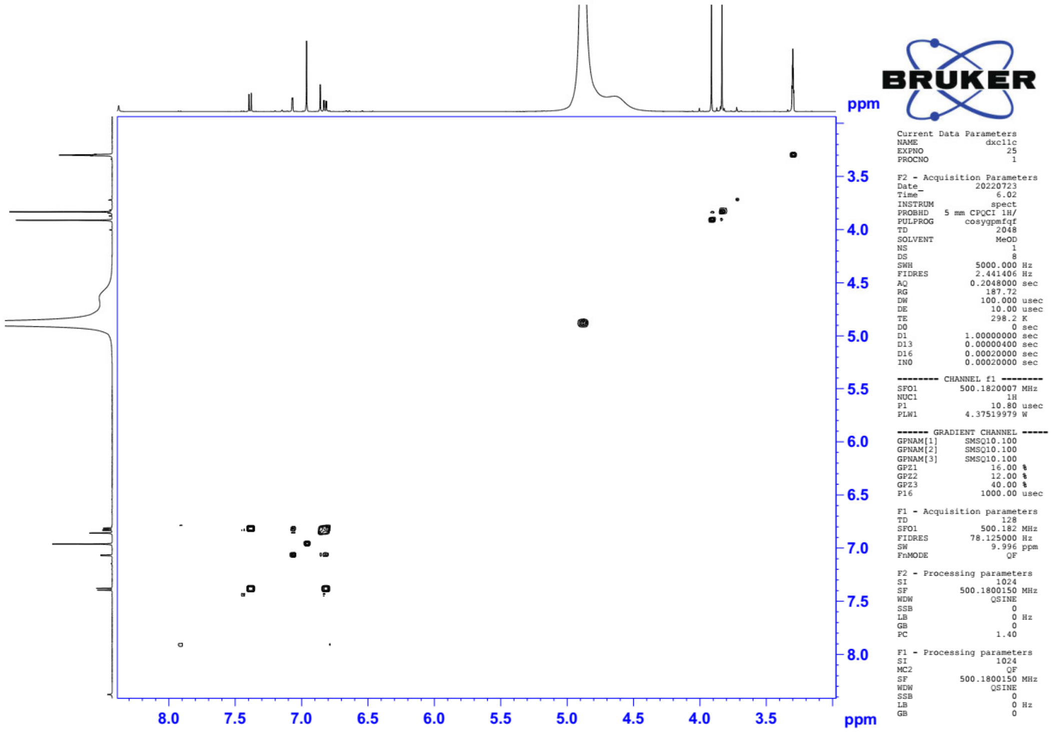

Figure S4-3. ^{1}H-^{1}H COSY spectrum of compound **4**.

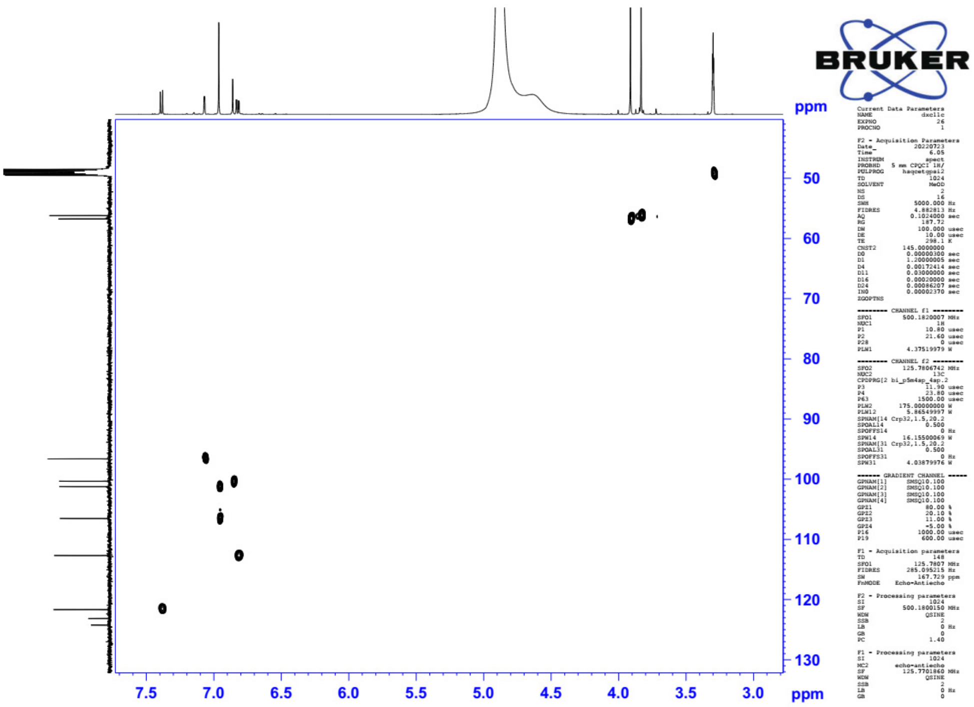

Figure S4-4. HSQC spectrum of compound **4**.

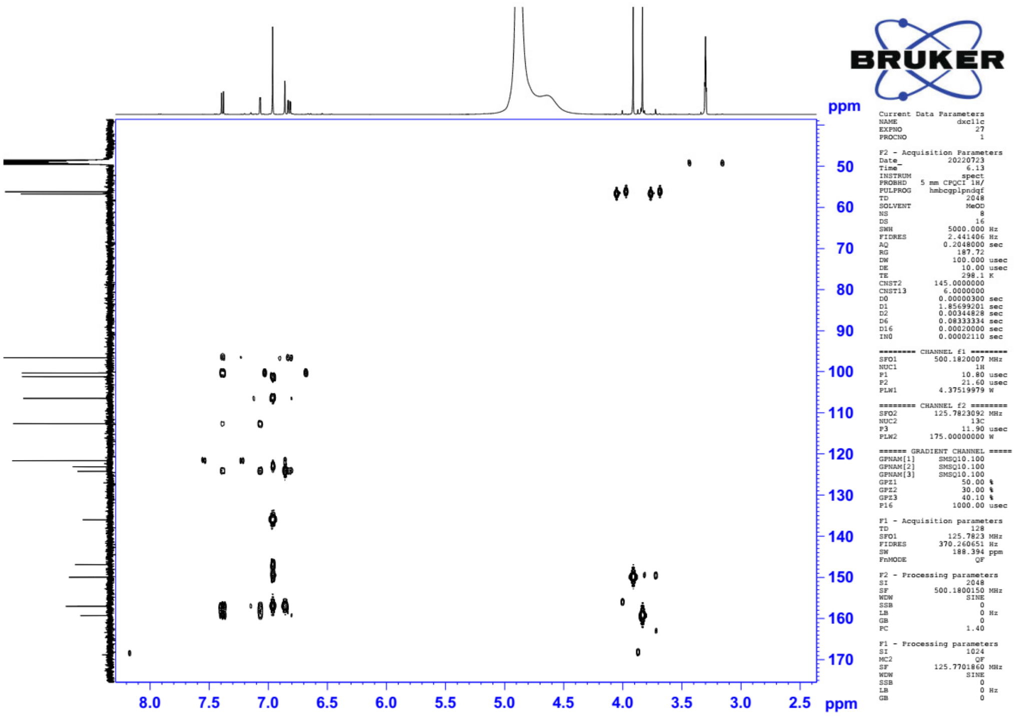

Figure S4-5. HMBC spectrum of compound **4**.

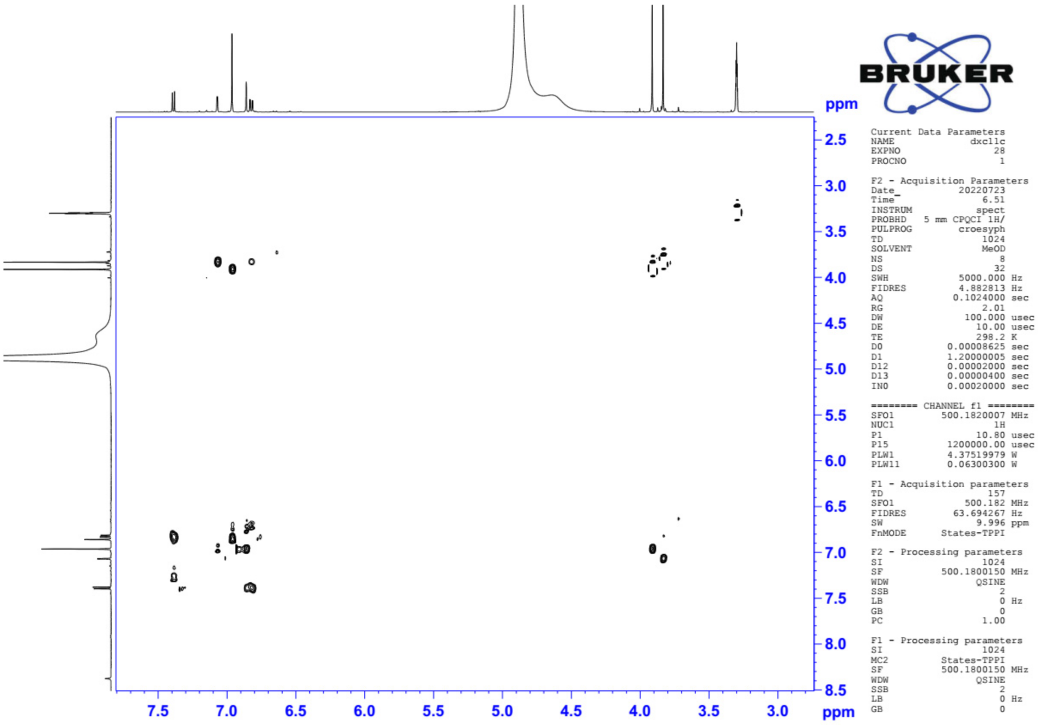

Figure S4-6. ROESY spectrum of compound **4**.

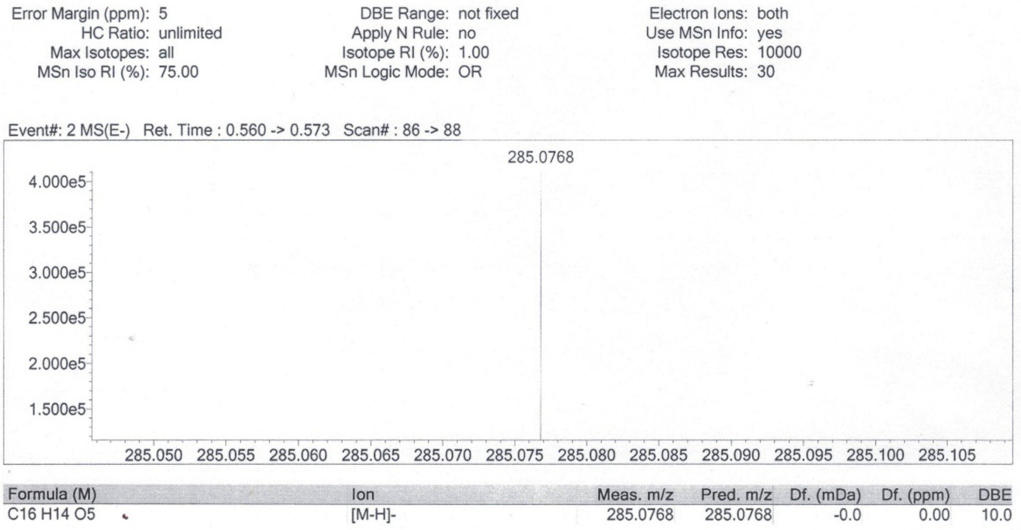

Figure S4-7. **(-)**-HRESIMS of compound **4**.

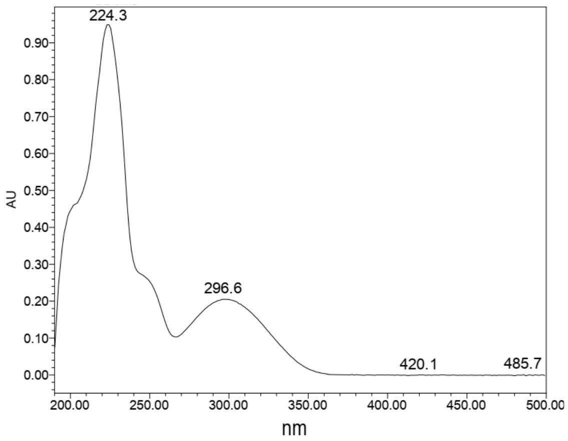

Figure S4-8. UV spectra of compound **4**.

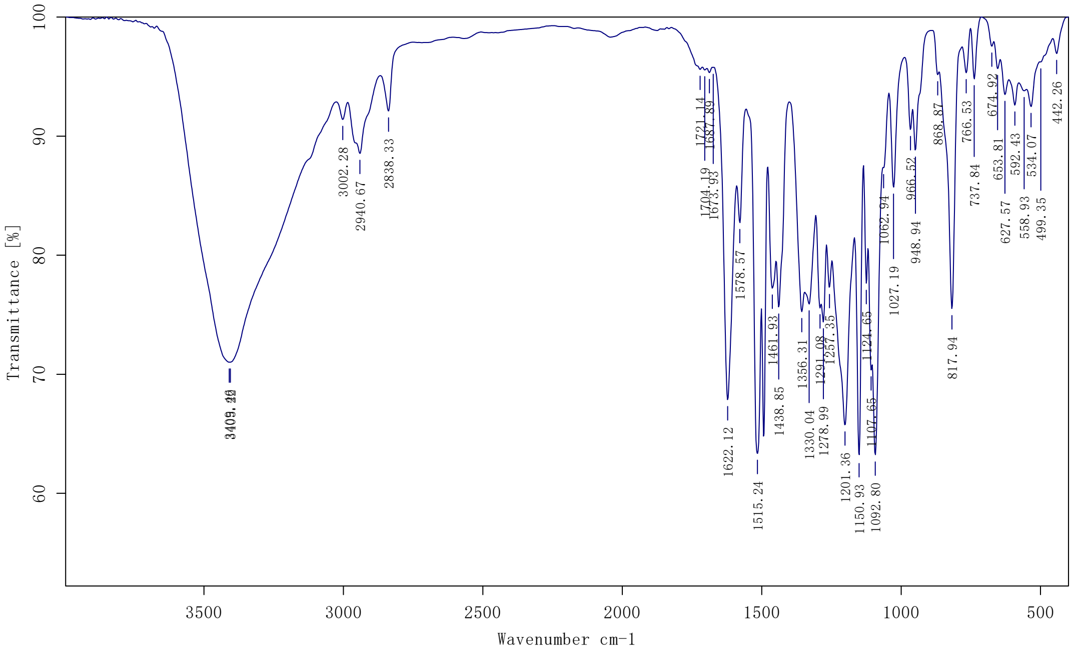

Figure S4-9. IR spectra of compound **4**.

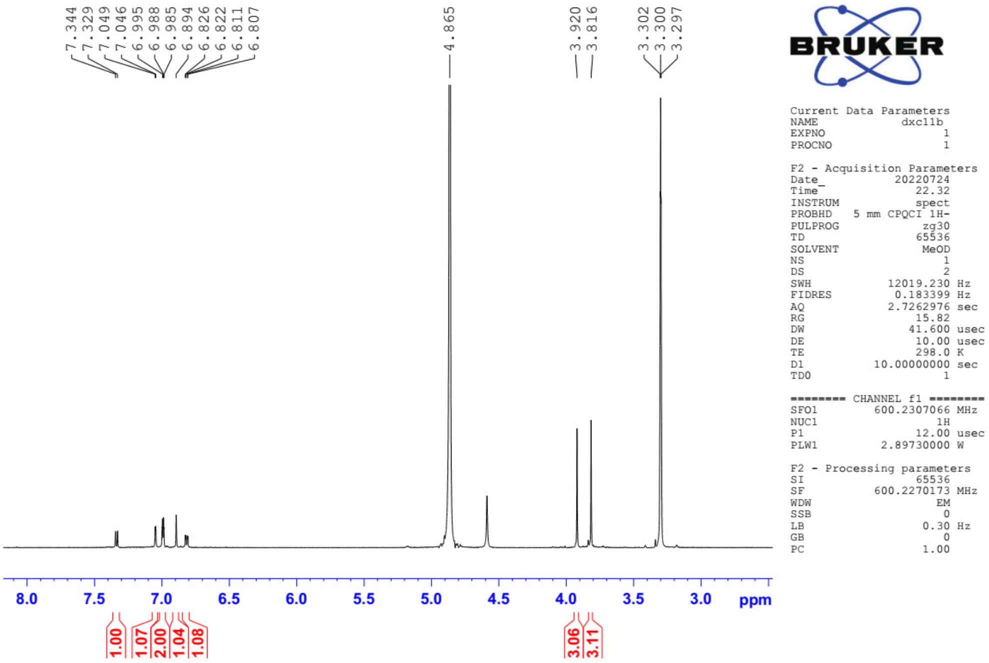

Figure S5-1. ^{1}H NMR (500 MHz, CD_3OD) spectrum of compound **5**.

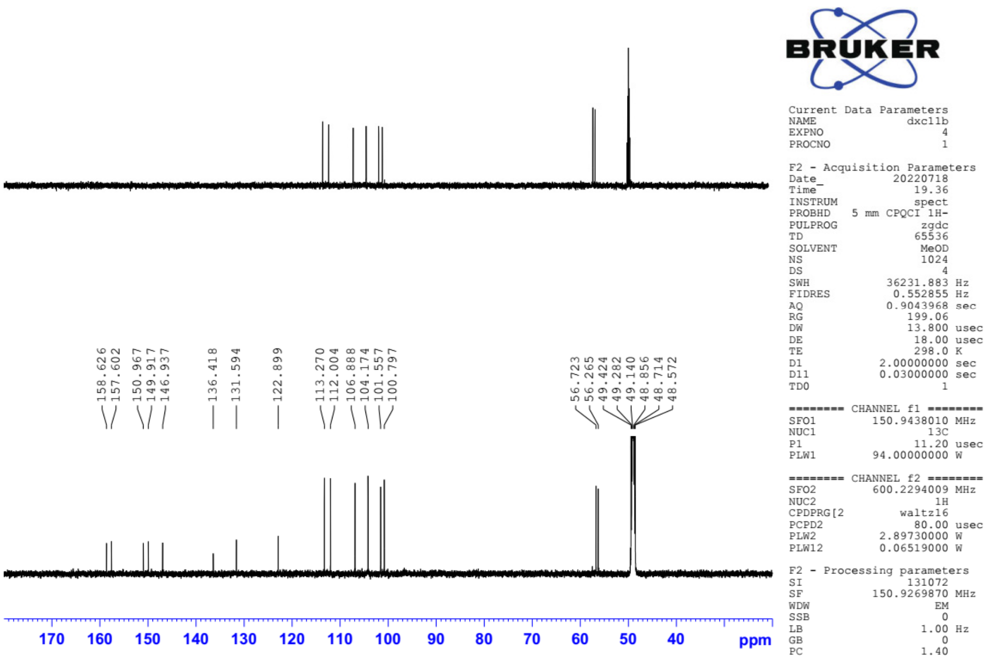

Figure S5-2. ^{13}C NMR and DEPT (125 MHz, CD_3OD) spectrum of compound **5**.

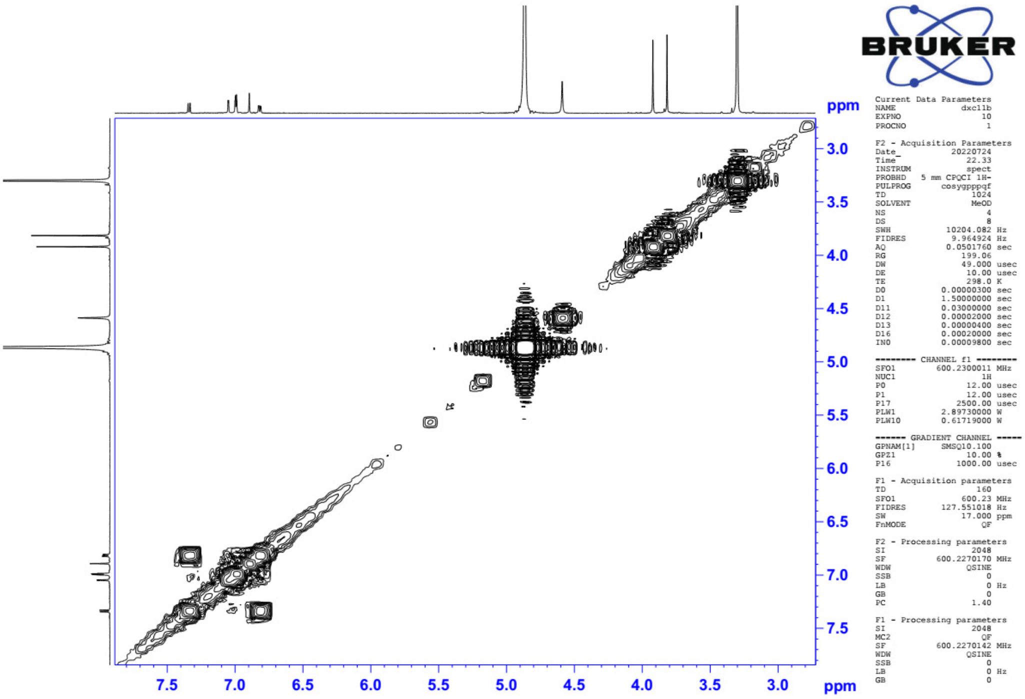

Figure S5-3. ^{1}H-^{1}H COSY spectrum of compound **5**.

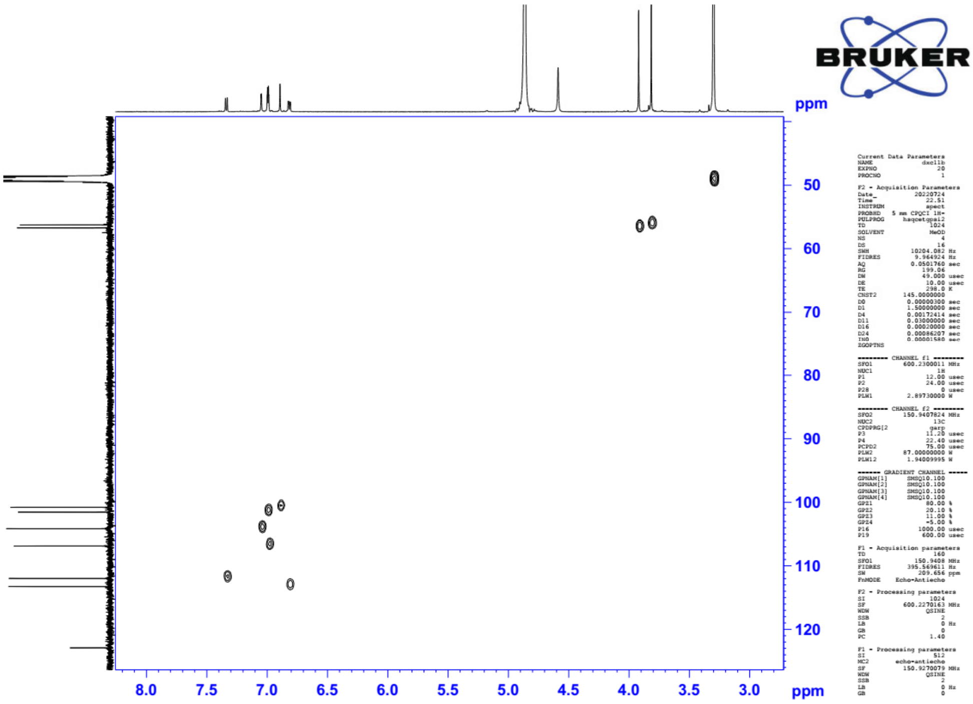

Figure S5-4. HSQC spectrum of compound **5**.

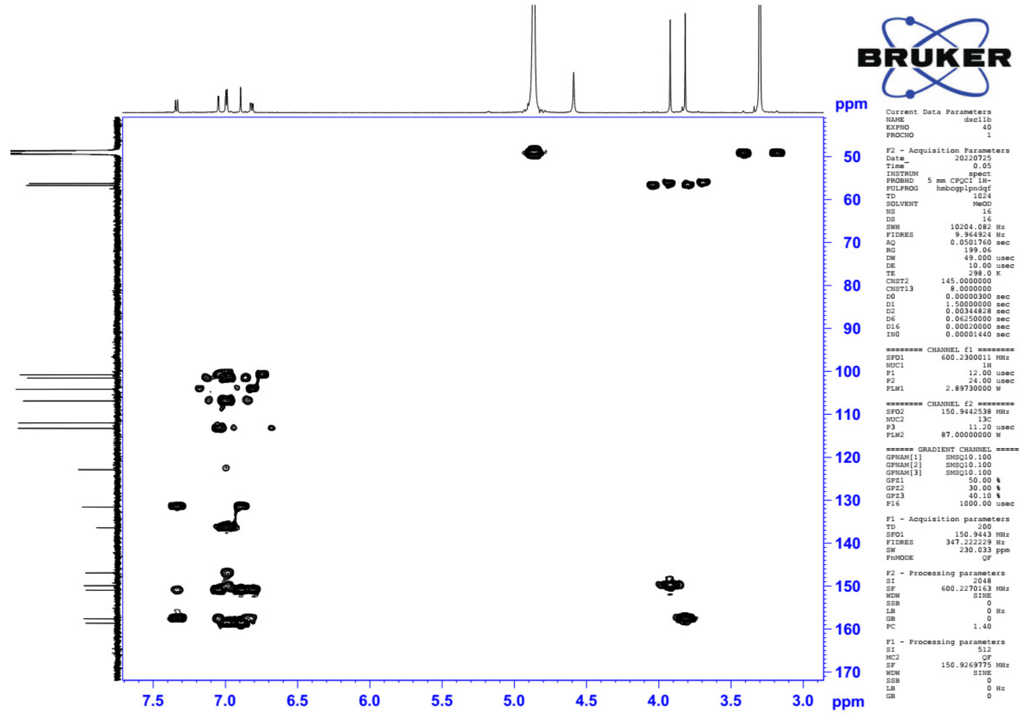

Figure S5-5. HMBC spectrum of compound **5**.

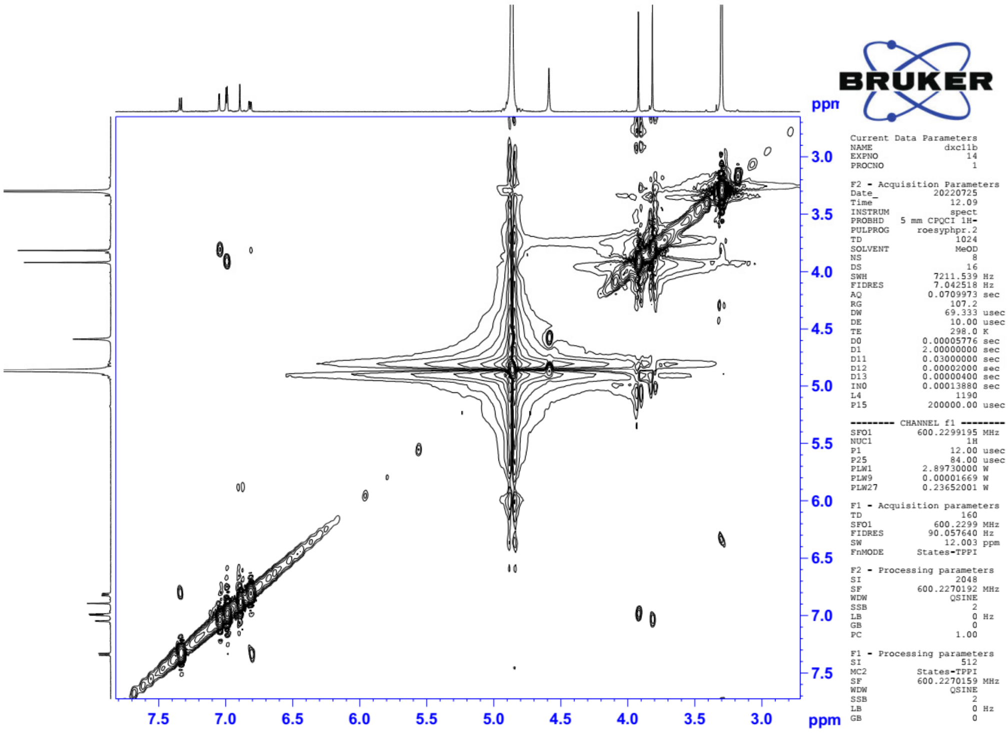

Figure S5-6. ROESY spectrum of compound **5**.

Error Margin (ppm):	5	DBE Range:	not fixed	Electron Ions:	both
HC Ratio:	unlimited	Apply N Rule:	no	Use MSn Info:	yes
Max Isotopes:	all	Isotope RI (%):	1.00	Isotope Res:	10000
MSn Iso RI (%):	75.00	MSn Logic Mode:	OR	Max Results:	30

Event#: 2 MS(E-) Ret. Time : 0.360 Scan# : 56

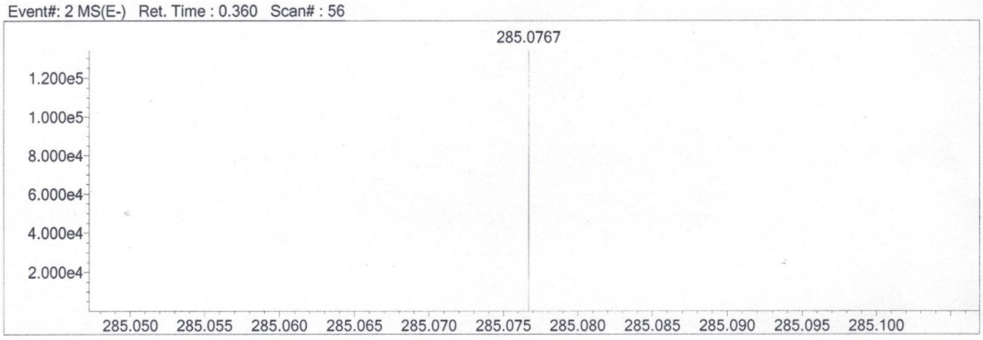

Formula (M)	Ion	Meas. m/z	Pred. m/z	Df. (mDa)	Df. (ppm)	DBE
C16 H14 O5	[M-H]-	285.0767	285.0768	-0.1	-0.35	10.0

Figure S5-7. (-)-HRESIMS of compound **5**.

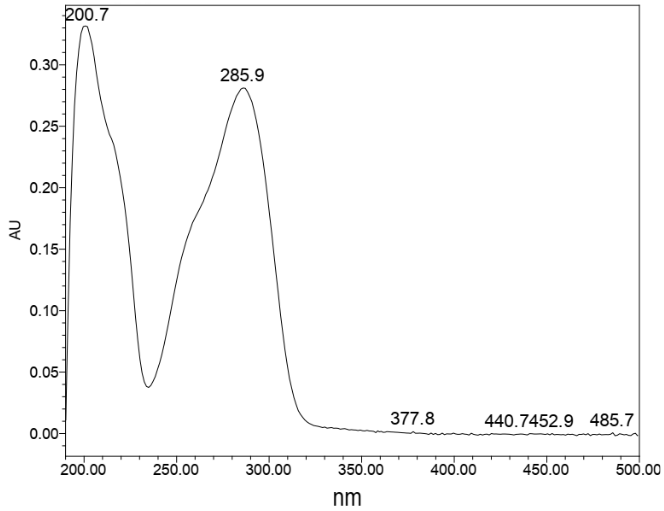

Figure S5-8. UV spectra of compound **5**.

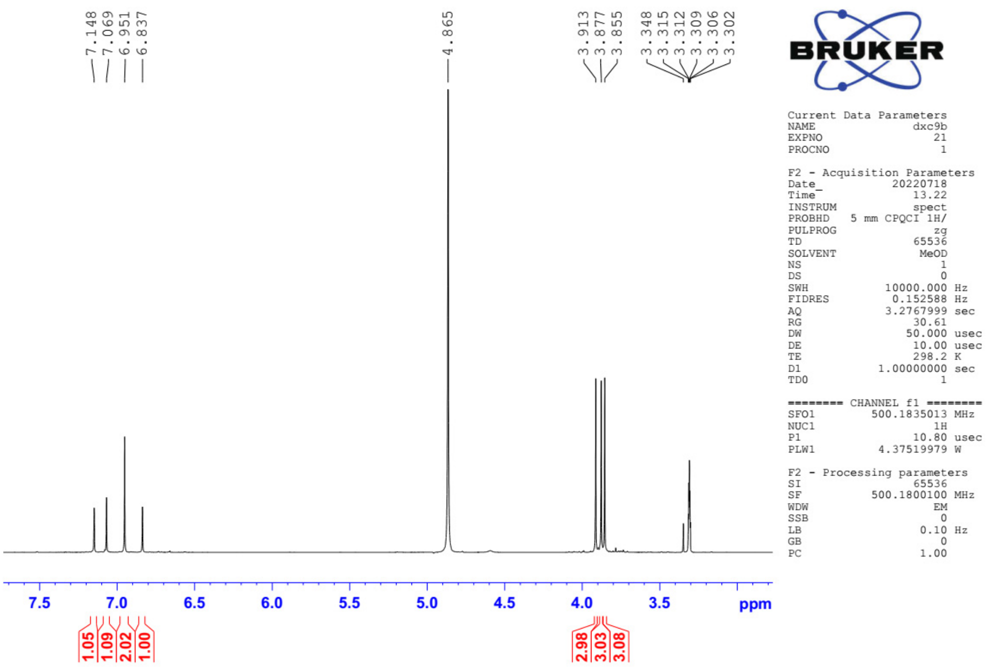

Figure S6-1. ^{1}H NMR (600 MHz, CD_3OD) spectrum of compound **6**.

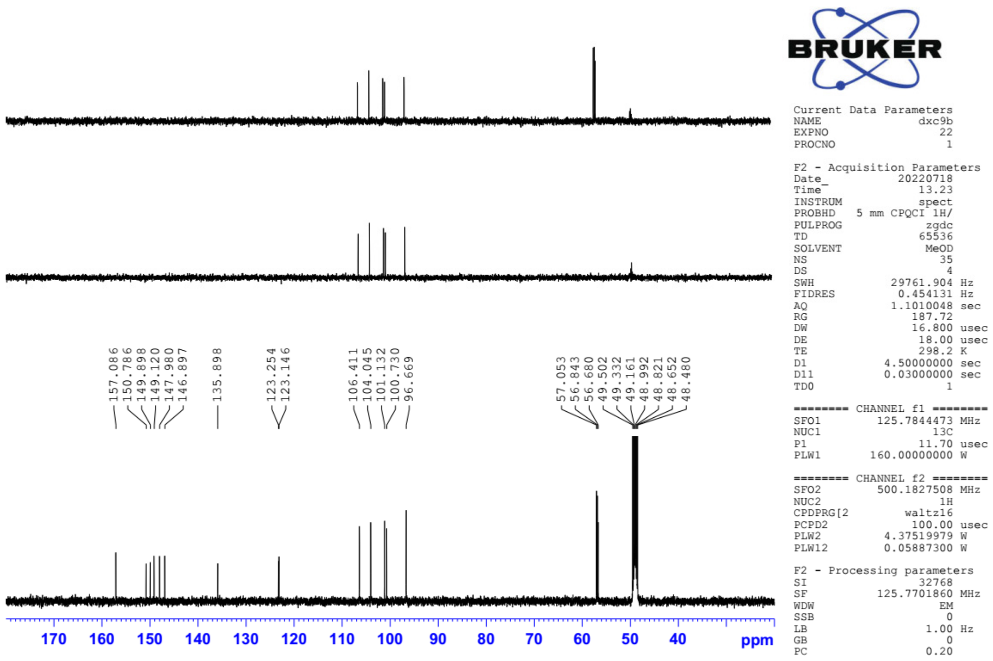

Figure S6-2. ^{13}C NMR and DEPT (150 MHz, CD_3OD) spectrum of compound **6**.

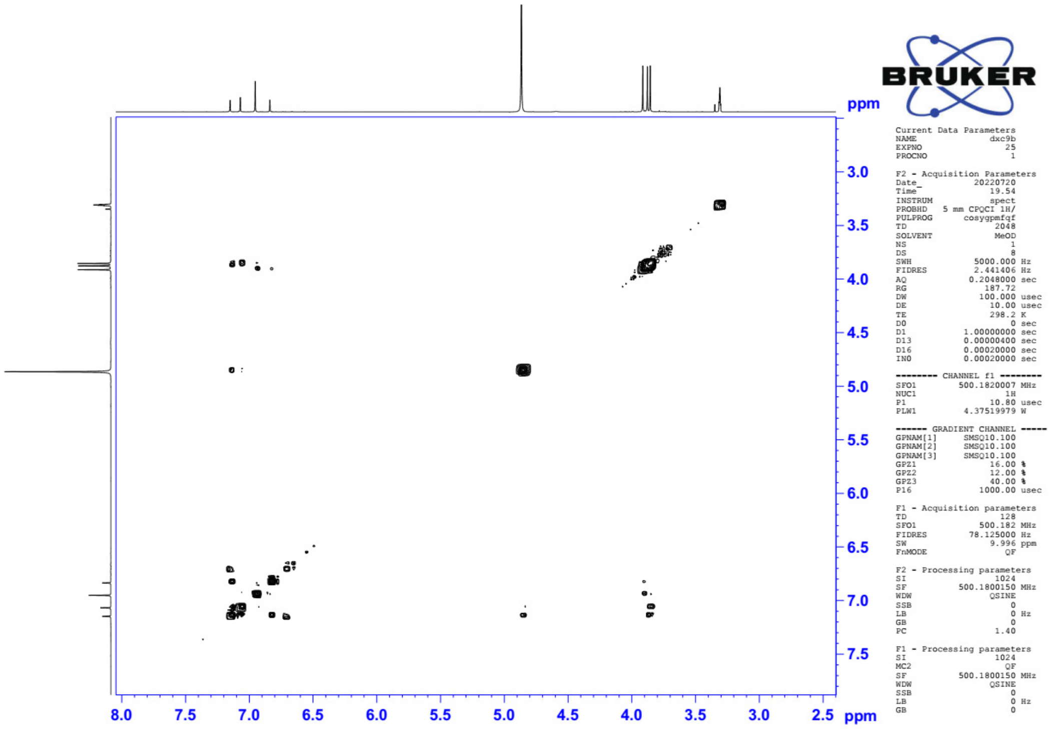

Figure S6-3. ^{1}H-^{1}H COSY spectrum of compound **6**.

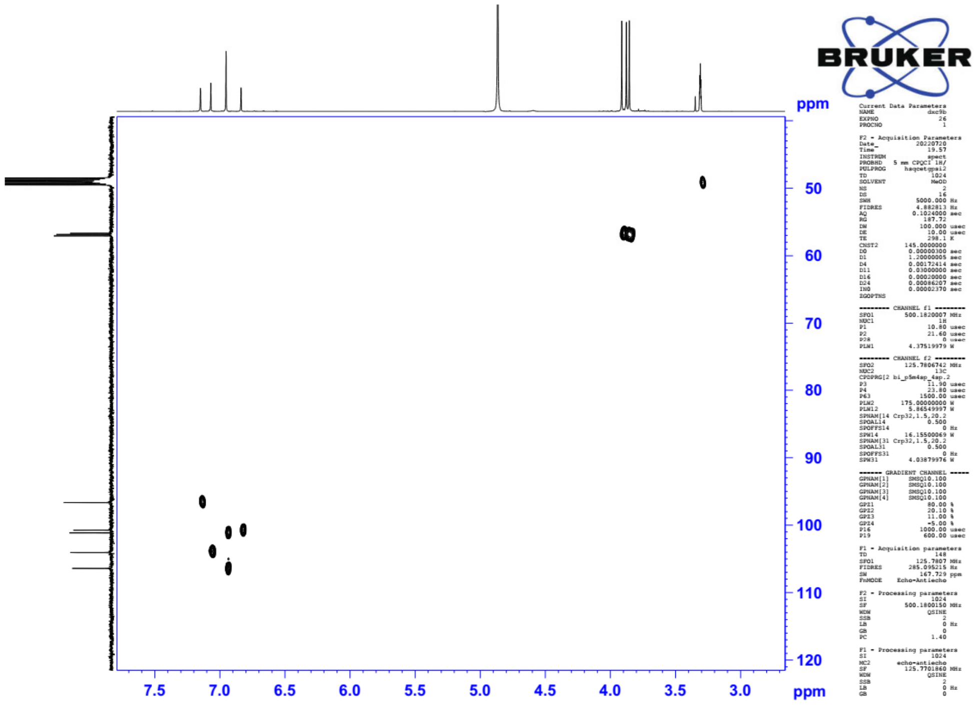

Figure S6-4. HSQC spectrum of compound **6**.

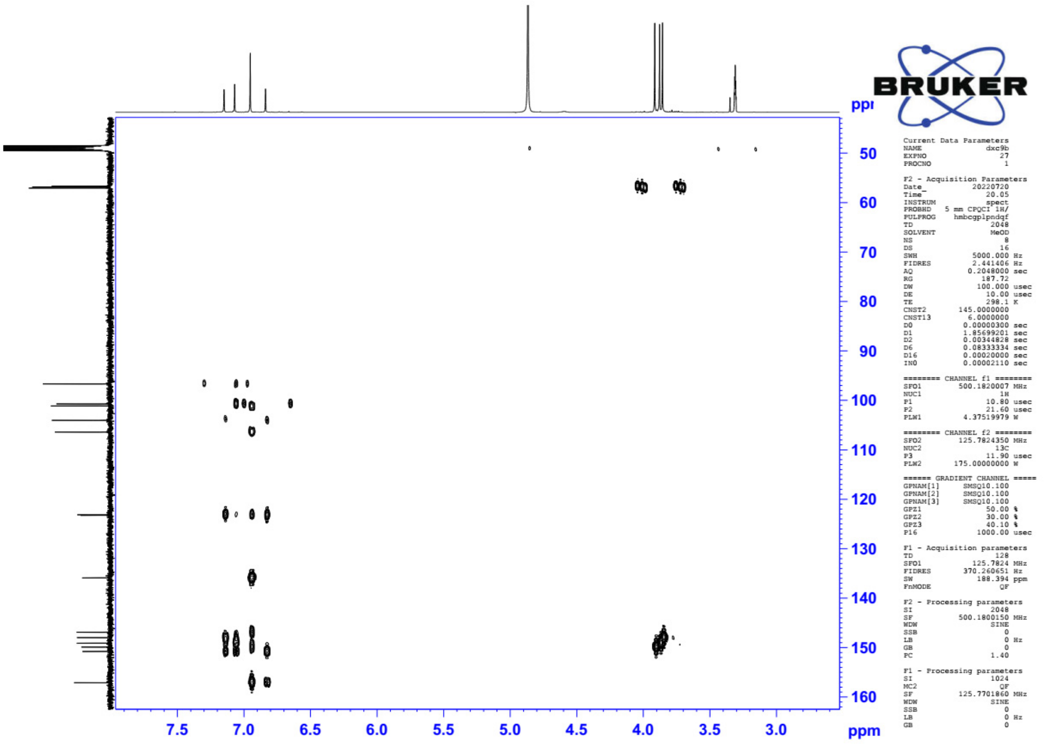

Figure S6-5. HMBC spectrum of compound **6**.

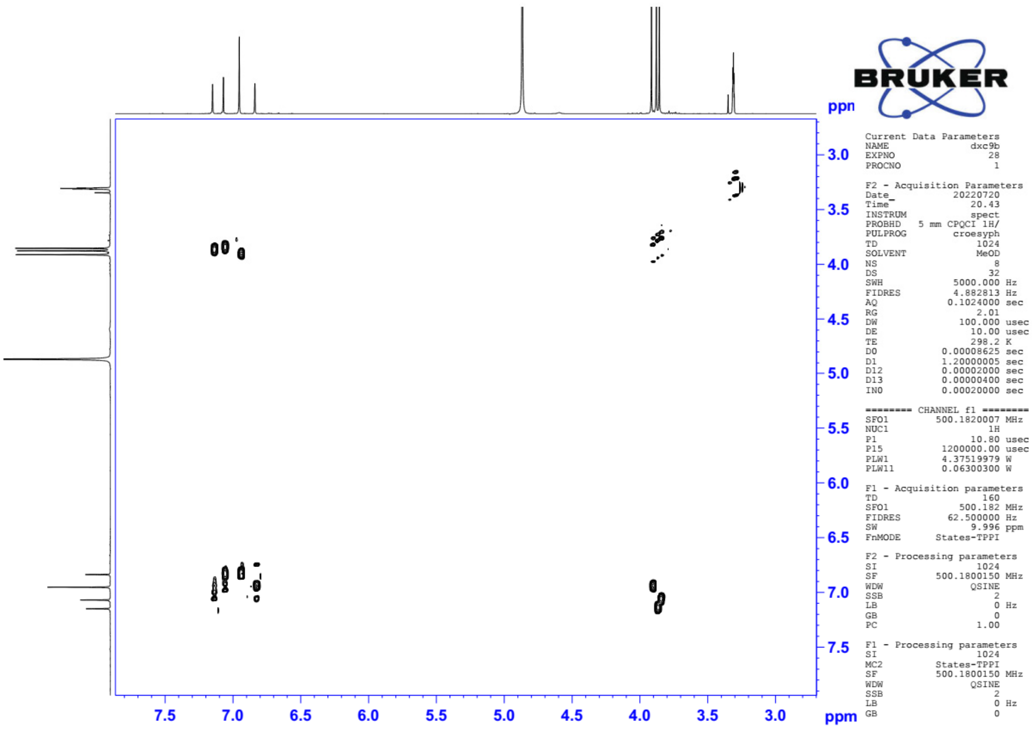

Figure S6-6. ROESY spectrum of compound **6**.

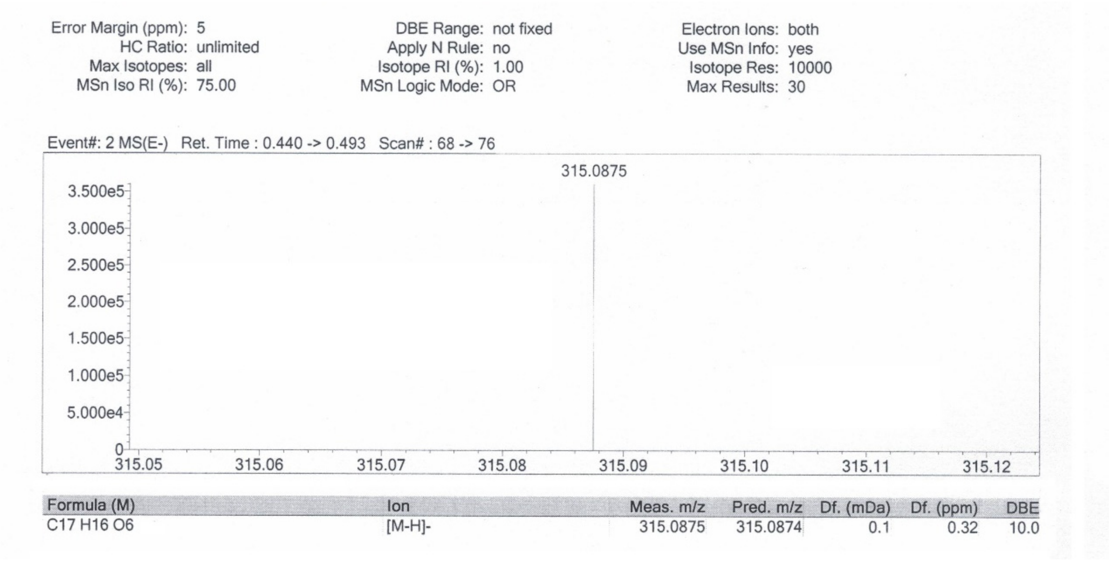

Figure S6-7. (-)-HRESIMS of compound **6**.

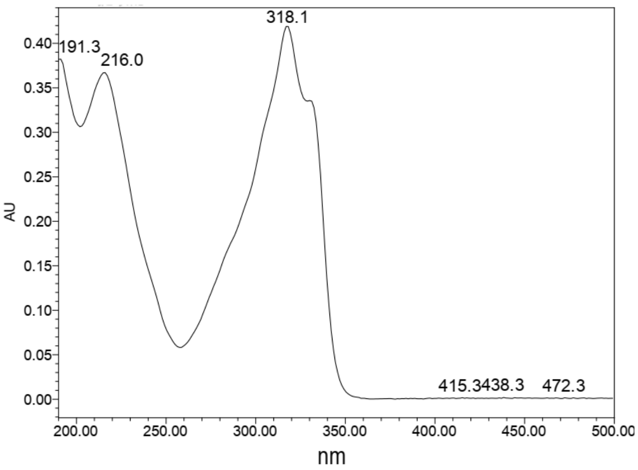

Figure S6-8. UV spectra of compound **6**.

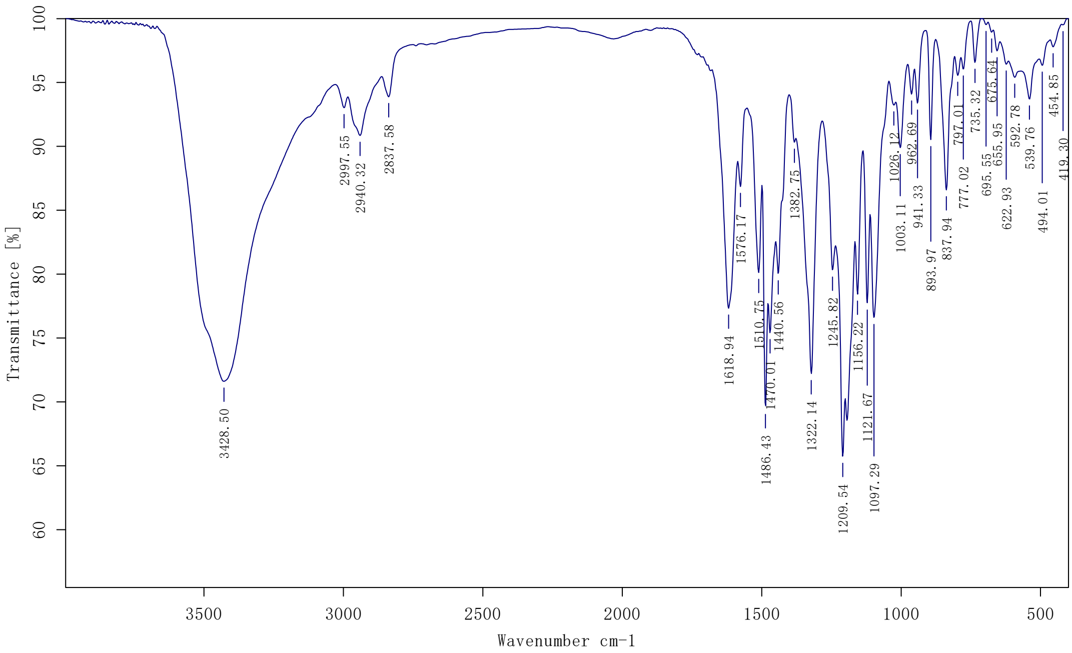

Figure S6-9. IR spectra of compound **6**.

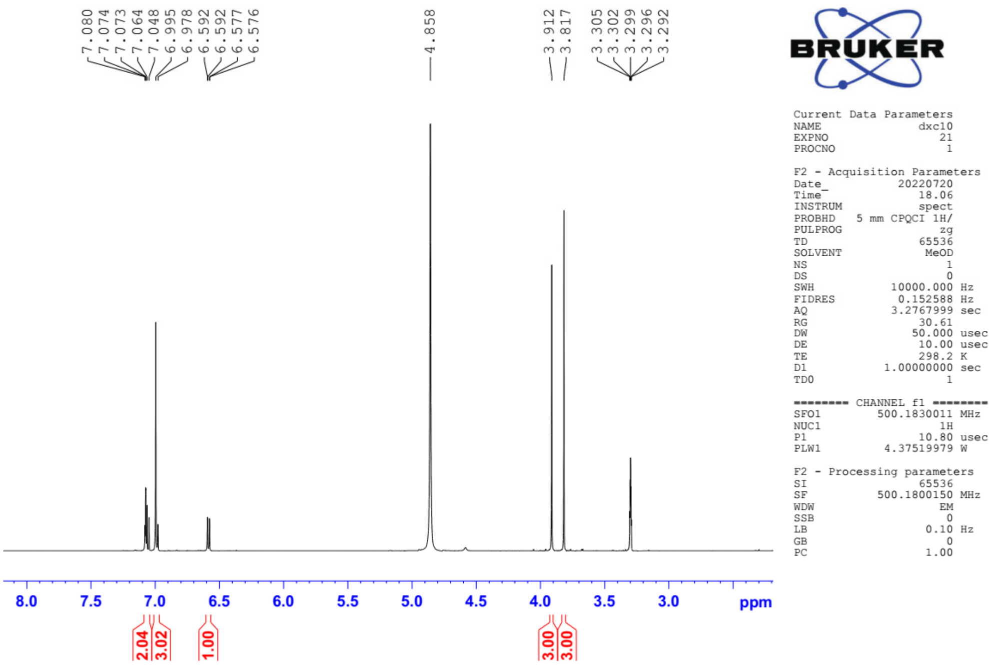

Figure S7-1. ^{1}H NMR (500 MHz, CD_3OD) spectrum of compound **7**.

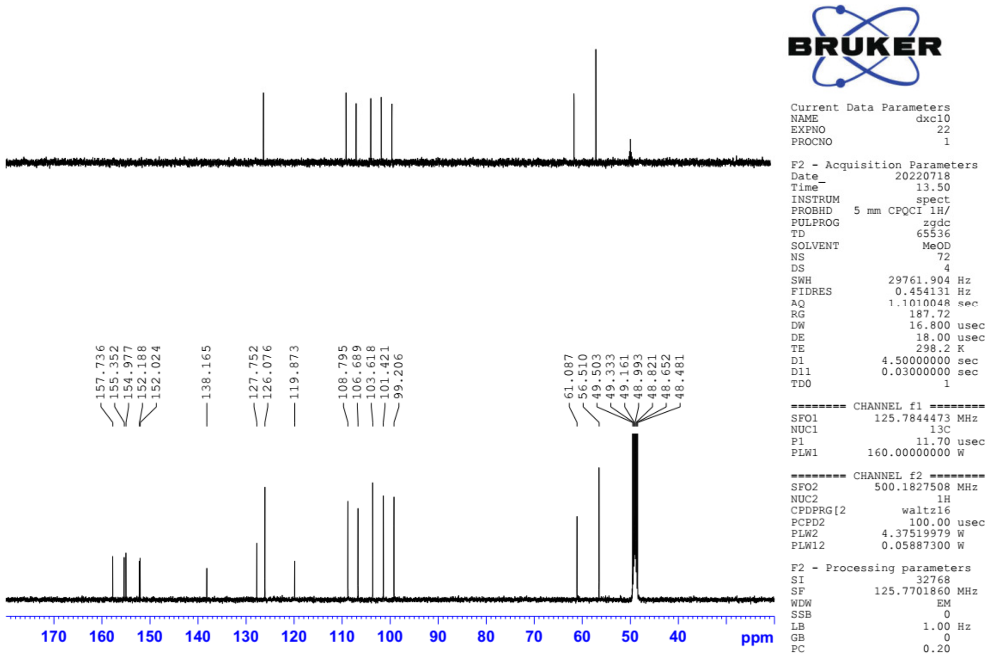

Figure S7-2. ^{13}C NMR and DEPT (125 MHz, CD_3OD) spectrum of compound **7**.

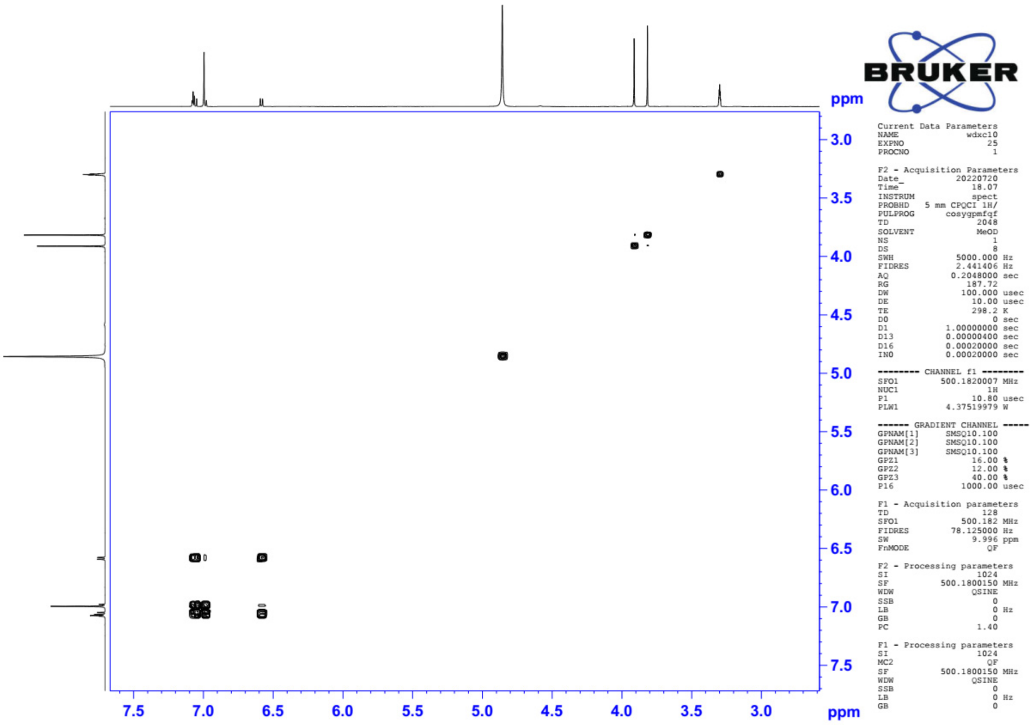

Figure S7-3. ^{1}H-^{1}H COSY spectrum of compound **7**.

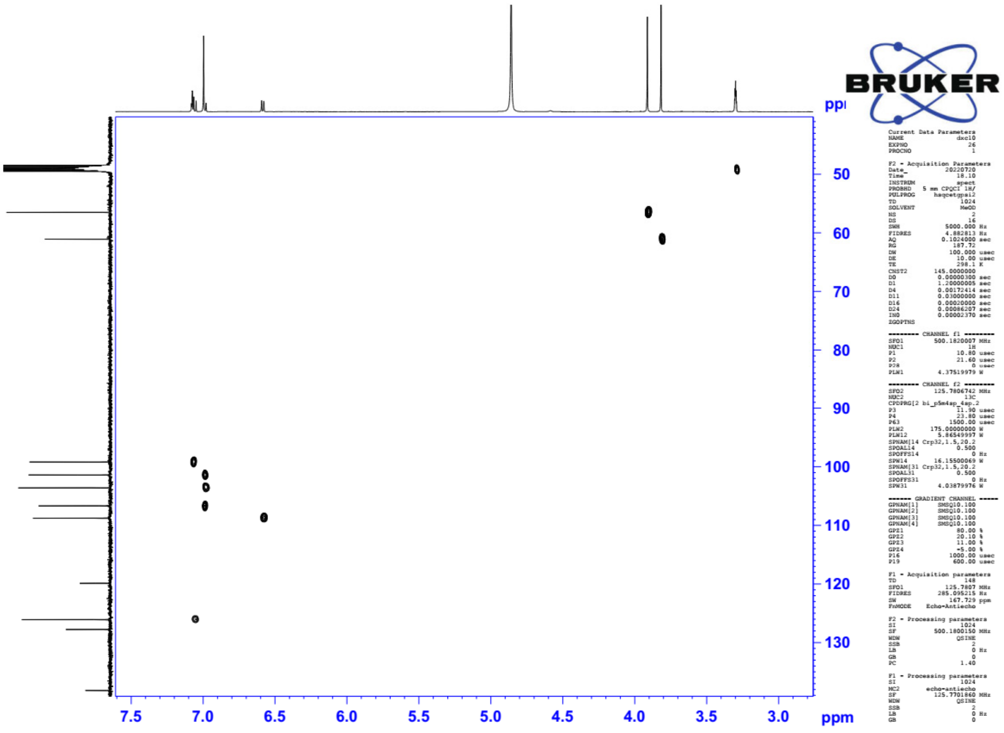

Figure S7-4. HSQC spectrum of compound **7**.

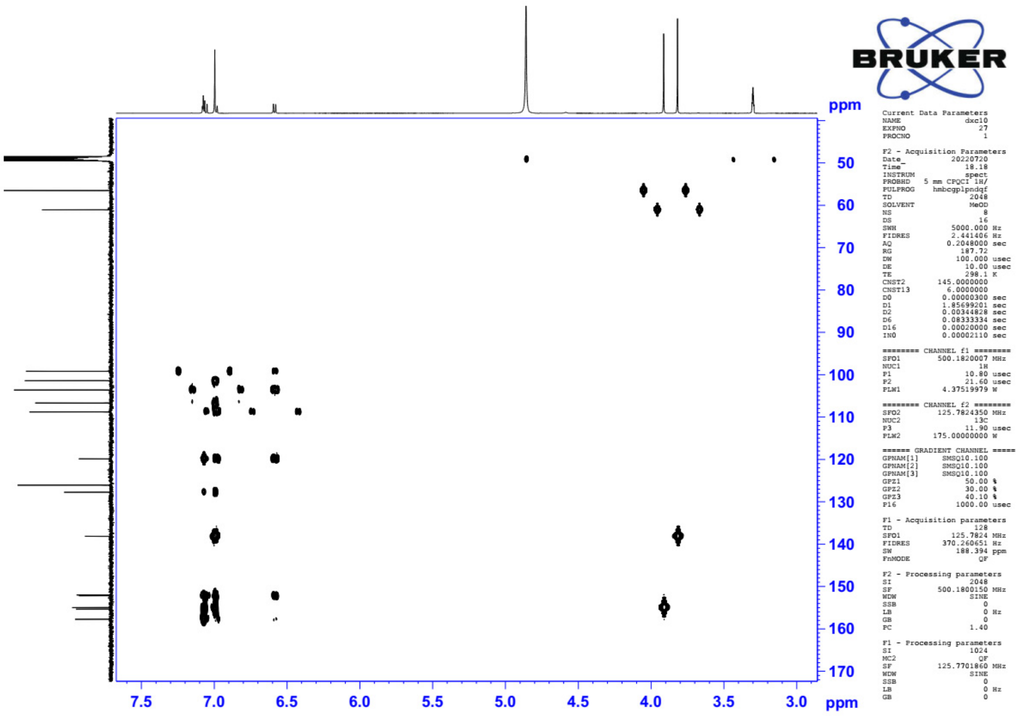

Figure S7-5. HMBC spectrum of compound **7**.

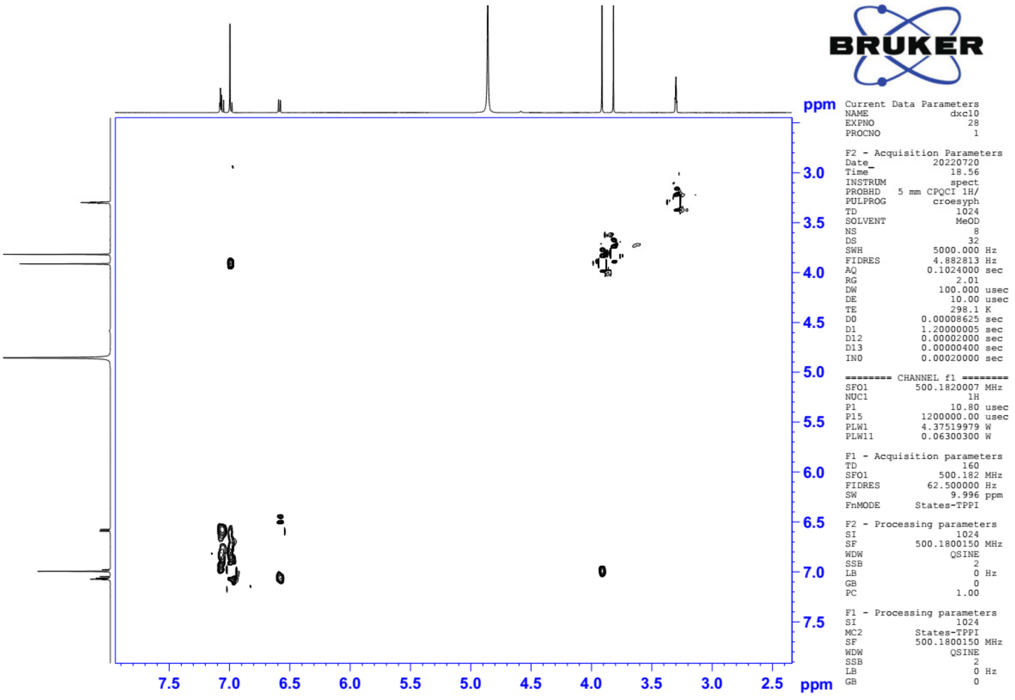

Figure S7-6. ROESY spectrum of compound **7**.

Error Margin (ppm):	5	DBE Range:	not fixed	Electron Ions:	both
HC Ratio:	unlimited	Apply N Rule:	no	Use MSn Info:	yes
Max Isotopes:	all	Isotope RI (%):	1.00	Isotope Res:	10000
MSn Iso RI (%):	75.00	MSn Logic Mode:	OR	Max Results:	30

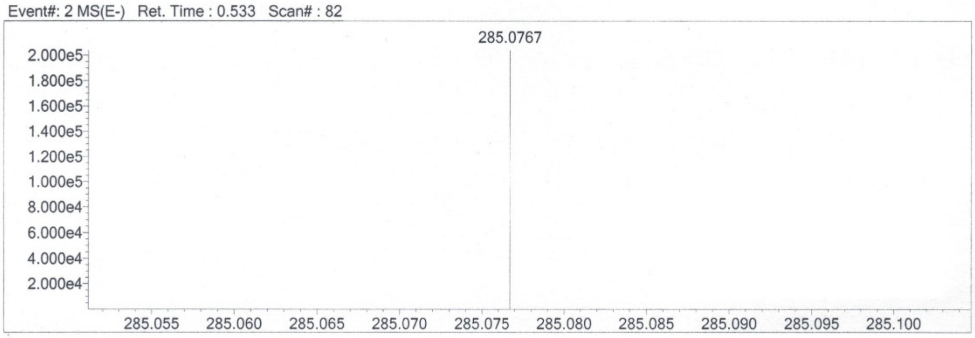

Formula (M)	Ion	Meas. m/z	Pred. m/z	Df. (mDa)	Df. (ppm)	DBE
C16 H14 O5	[M-H]-	285.0767	285.0768	-0.1	-0.35	10.0

Figure S7-7. (-)-HRESIMS of compound **7**.

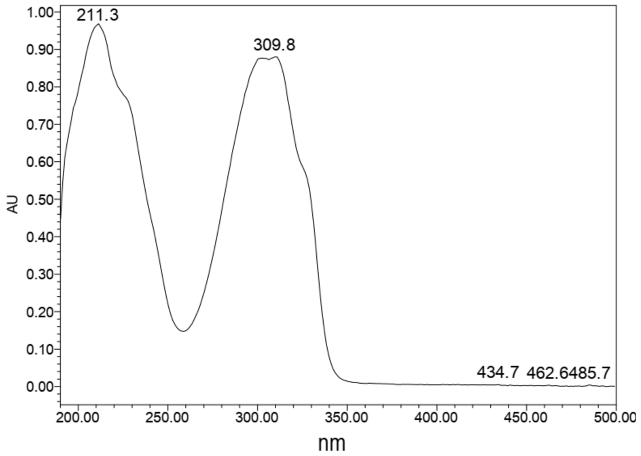

Figure S7-8. UV spectra of compound **7**.

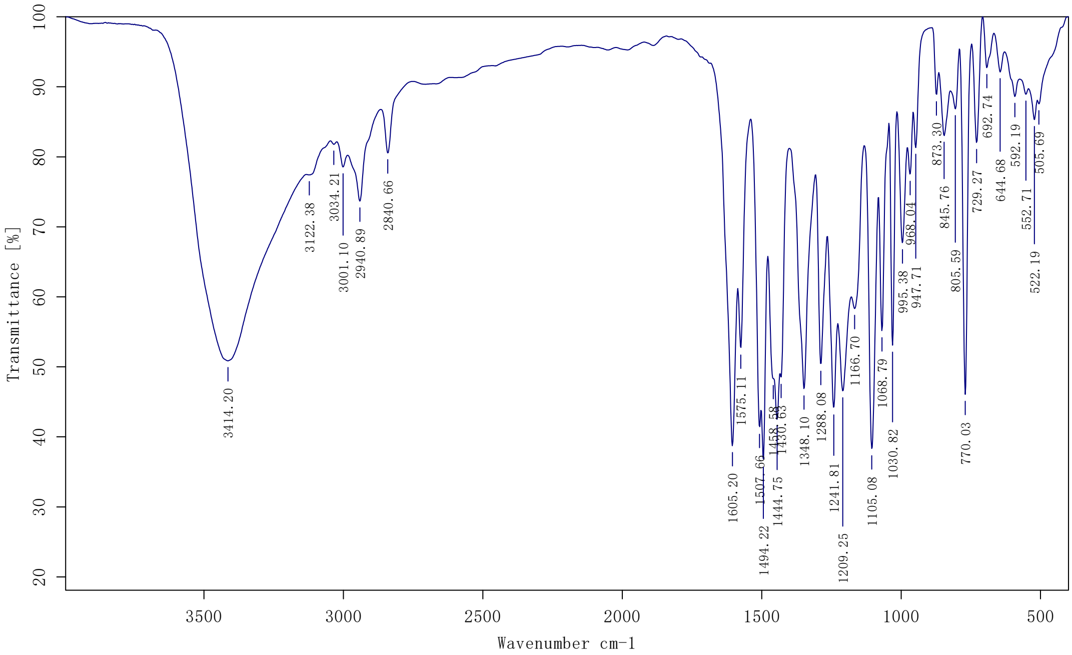

Figure S7-9. IR spectra of compound **7**.

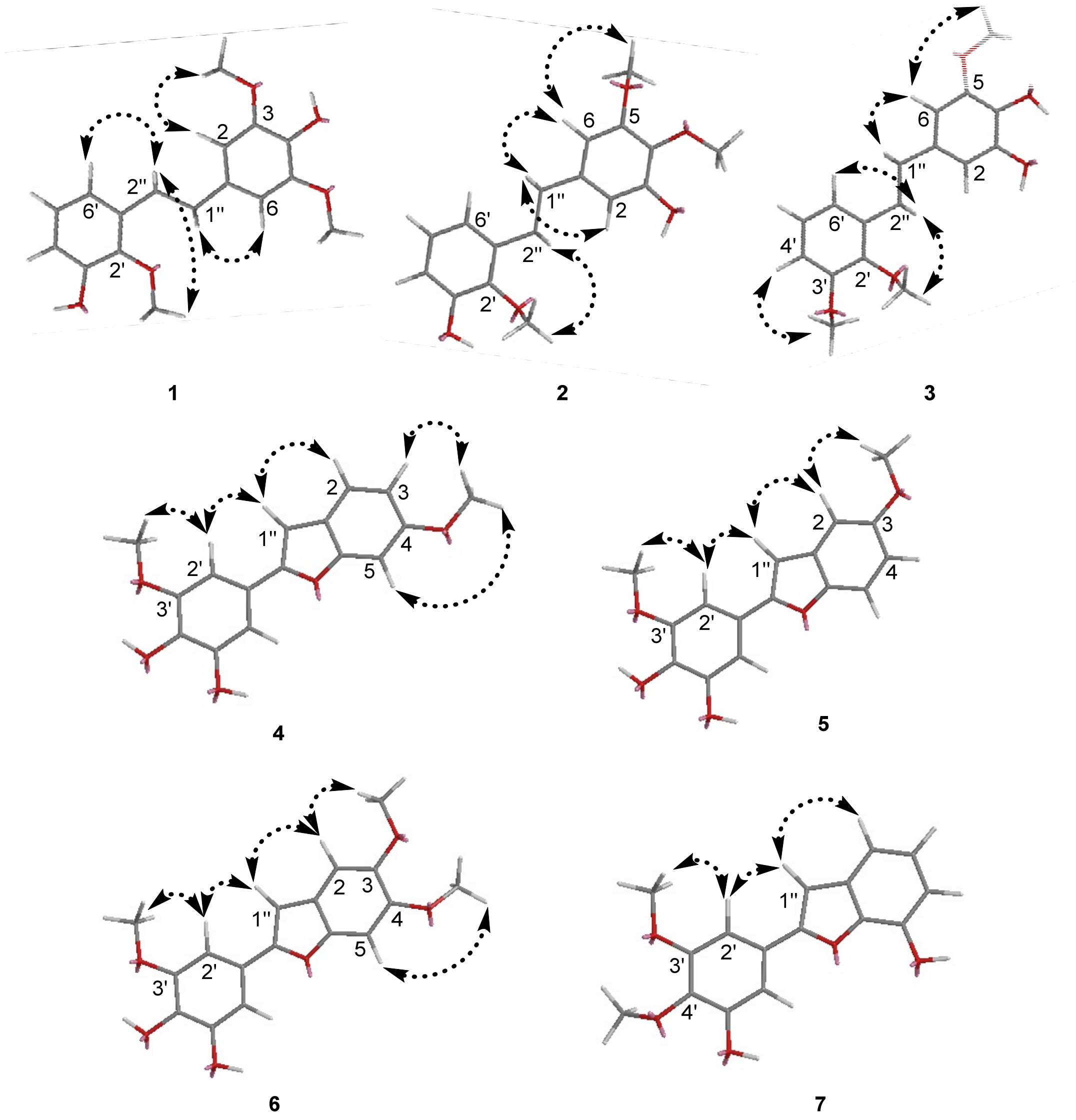

Figure S8. ROESY correlations of compounds **1**-**7**.
